# Fujian cytoplasmic male sterility and the fertility restorer gene *OsRf19* provide a promising breeding system for hybrid rice

**DOI:** 10.1073/pnas.2208759119

**Published:** 2022-08-15

**Authors:** Haichao Jiang, Qing Lu, Shuqing Qiu, Huihui Yu, Zhengji Wang, Zhichao Yu, Yunrui Lu, Lei Wang, Fan Xia, Yuying Wu, Fan Li, Qinglu Zhang, Gang Liu, Dingding Song, Chonglie Ma, Qi Ding, Xiaobo Zhang, Lin Zhang, Xuetang Zhang, Xu Li, Jianwei Zhang, Jinghua Xiao, Xianghua Li, Naiyuan Wang, Yidan Ouyang, Fasong Zhou, Qifa Zhang

**Affiliations:** ^a^National Key Laboratory of Crop Genetic Improvement, Hubei Hongshan Laboratory, Huazhong Agricultural University, 430070 Wuhan, China;; ^b^State Key Laboratory of Crop Breeding Technology Innovation and Integration, Life Science and Technology Center, China National Seed Group Co., Ltd, Wuhan, 430074, China

**Keywords:** rice, cytoplasmic male sterility, restorer of fertility, *OsRf19*, hybrid rice breeding

## Abstract

Cytoplasmic male sterility (CMS) determined by mitochondrial genes and restorer of fertility (*Rf*) controlled by nuclear-encoded genes provide the breeding systems of many hybrid crops for the utilization of heterosis. Although several CMS/*Rf* systems have been widely exploited in rice, hybrid breeding using these systems has encountered difficulties due to either fertility instability or complications of two-locus inheritance or both. In this work, we characterized a type of CMS, Fujian Abortive cytoplasmic male sterility (CMS-FA), with stable sporophytic male sterility and a nuclear restorer gene that completely restores hybrid fertility. CMS is caused by the chimeric open reading frame *FA182* that specifically occurs in the mitochondrial genome of CMS-FA rice. The restorer gene *OsRf19* encodes a pentatricopeptide repeat (PPR) protein targeted to mitochondria, where it mediates the cleavage of *FA182* transcripts, thus restoring male fertility. Comparative sequence analysis revealed that *OsRf19* originated through a recent duplication in wild rice relatives, sharing a common ancestor with *OsRf1a*/*OsRf5*, a fertility restorer gene for Boro II and Hong-Lian CMS. We developed six restorer lines by introgressing *OsRf19* into parental lines of elite CMS-WA hybrids; hybrids produced from these lines showed equivalent or better agronomic performance relative to their counterparts based on the CMS-WA system. These results demonstrate that CMS-FA/*OsRf19* provides a highly promising system for future hybrid rice breeding.

## Significance

Although hybrid rice has been widely utilized for nearly half a century, tremendously improving rice productivity worldwide, the breeding of hybrids has been difficult because of genetic complications in male sterility and fertility-restoring systems currently available in rice. Here, we characterized Fujian Abortive cytoplasmic male sterility (CMS-FA) rice, which has shown stable male sterility controlled by the mitochondrial gene *FA182*; a single nuclear gene, *OsRf19*, completely restores fertility. This single-gene inheritance has greatly eased the breeding process. By converting CMS-WA hybrids with the CMS-FA system, we developed six hybrids that showed equivalent or better performance relative to their CMS-WA counterparts. CMS-FA/*OsRf19* provides a promising system for future hybrid rice breeding.

Cytoplasmic male sterility (CMS), a maternally inherited trait characterized by a lack of functional pollen, is a widespread phenomenon observed in more than 200 species of higher plants (1). CMS is usually determined by the mitochondrial genome but can be suppressed or counteracted by nuclear genes known as restorer of fertility (*Rf*). The CMS/*Rf* system is widely used in commercial production of hybrid seeds of many crops and also provides an excellent model for studying mitochondrial–nuclear interaction and coevolution in plants. CMS hybrid rice has been successfully developed with a three-line system containing a male sterile line, a maintainer line, and a restorer line, which has contributed greatly to food production worldwide since the 1970s.

Several types of CMS systems have been identified in rice, including CMS-BT (2, 3), CMS-HL (4), CMS-LD (5, 6), CMS-CW (7, 8), CMS-WA (9), CMS-RT102 (10), CMS-RT98 (11), CMS-D1 (12), and CMS-TA (13, 14). Each of the above CMS systems is associated with a chimeric open reading frame (ORF) that is thought to originate from mitochondrial genomic rearrangement (15). For example, *orf79* for CMS-BT and *orfH79* for CMS-HL share 98% identity at the nucleotide level and encode small proteins with an N terminus similar to COX1 (3, 4). The *orf307* gene for CMS-CW contains the 5′ sequence of *orf288* and a sequence of unknown origin (7, 8). The *orf352* gene for CMS-RT102 is cotranscribed with the ribosomal protein gene *rpl5*, and the 2.8-kb *rpl5–orf352* transcripts are processed into 2.6-kb transcripts with a cleavage inside the *orf352* coding region in the presence of the *Rf* gene (10). The *orf113* gene for CMS-RT98 is cotranscribed with *atp4* and *cox3* encoding adenosine 5′-triphosphate (ATP) synthase F0 subunit 4 and Cyt *c* oxidase subunit 3, respectively, and their transcripts are distinctly processed in the presence of a fertility restorer gene (11). The CMS-WA gene *WA352* comprises three segments derived from the putative mitochondrial ORFs *orf284*, *orf224*, and *orf288* and a short sequence of unknown origin (9). *WA314* is another CMS gene found in CMS-WA that causes partial male sterility in transgenic plants (16). The CMS-D1 gene *orf182* is composed of three recombinant fragments, the largest of which has high similarity to the *Sorghum bicolor* mitochondrial sequence (12). The *orf312* gene for CMS-TA is similar to the previously described *orf288*, a part of which is among the components comprising *WA352*, a chimeric CMS-associated gene of CMS-WA (13).

Most of the cloned *Rf* genes belong to a large, well-defined family of genes that encode organelle-targeted pentatricopeptide repeat (PPR) proteins. To date, five *Rfs* have been cloned and characterized as PPR proteins in rice: *Rf1a* and *Rf1b* in CMS-BT rice (2, 3), *Rf5* and *Rf6* in CMS-HL rice (17, 18), and *Rf4* in CMS-WA rice (19, 20). *Rfo* in *Brassica napus* Ogura-CMS (21), *Rfp* in *Brassica napus pol*-CMS (22), *Rfh* in *Brassica napus hau*-CMS (23), and *Rf3* in maize CMS-S (24) also encode PPR proteins. In addition to PPR proteins, other types of *Rfs* have been identified, including *Rf2* in CMS-LD encoding a glycine-rich protein (5) and *Rf17* encoding a mitochondrial-targeting protein containing a part of an acyl-carrier protein synthase-like domain in rice (25), as well as *Rf2* in maize CMS-T encoding an aldehyde dehydrogenase (26).

PPR proteins are characterized by the presence of a degenerate 35-amino acid repeat, the PPR motif, which is arranged in tandem 2 to 50 times (27). Several PPR proteins exhibit RNA-binding activity and have been implicated in posttranscriptional regulation in organelle (mitochondria and chloroplasts) gene expression, including RNA editing, processing, intron splicing, translation, and messenger RNA (mRNA) stability (28–30). In organelles of flowering plants, RNA editing results in alteration of the coding sequences in many of the organellar transcripts and produces translatable mRNAs by creating AUG start sites or eliminating premature stop codons. RNA editing may also affect the RNA structure, influence splicing, or alter the stability of RNAs (31–33).

Fujian Abortive (FA) CMS rice was bred using the cytoplasm from a common wild rice (*Oryza rufipogon* L.), which was found in Fujian Province, China (34–36). Similar to CMS-WA, the most widely used system in hybrid rice breeding worldwide, the male sterility of CMS-FA is sporophytic, with fertility determined by the parental genotype (37), which is a preferred system compared to gametophytic male sterility, in which fertility is determined by the pollen genotype. An effort to search for restorers by crossing the CMS-FA line Jinnong1A with 220 diverse rice varieties, including many restorer lines of the CMS-WA system, failed to find any restorer line (35). Therefore, CMS-FA is genetically distinct from CMS-WA with respect to CMS and fertility restoration genes. Moreover, the sterility-restoration of the CMS-FA/*Rf* progeny segregated as a single-locus inheritance (36, 37), which is also different from the two-locus inheritance of the CMS-WA system. Together, these two characteristics make CMS-FA more appealing for hybrid rice breeding than other currently widely used CMS systems such as CMS-WA and CMS-HL. A number of FA sterile lines and restorer lines have been bred and released for commercial use and have demonstrated superior performance in rice production, showing the promise for future hybrid rice breeding.

In the work reported here, we identified the chimeric ORF *orf182* in the mitochondrial genome of CMS-FA as the cause of male sterility and a gene encoding a PPR protein as the restorer gene. Considering the current situation in which CMS and fertility restorer genes have been identified from many species and all are named *orf* and *Rf* irrespective of their molecular nature, for clarity we suggest adding a prefix of the species name to each *Rf* gene as well as a prefix to specify the cytoplasm source for each CMS gene. We thus named this restorer gene *OsRf19* in sequence with other identified CMS-restorer genes in rice (https://archive.gramene.org/db/genes/search_gene?query=fertility+restoration&vocabulary=genes&search_box_name=query&search_box_id=gene_search_for&search_field=name&gene_type_id=&species=1&x=9&y=9) and the CMS gene *FA182*. We showed that OsRF19 restores fertility by cleaving/degrading *FA182* mRNA.

## Results

### The Anther Morphology of the CMS-FA Lines.

Both the maintainer line Shen95B and the male sterile line Shen95(FA)A of CMS-FA grew normally during the vegetative stage (*SI Appendix*, Fig. S1*A*). Shen95B has normal anthers that are plump and yellow, but the anthers of Shen95(FA)A are small, shrunken, and transparent (*SI Appendix*, Fig. S1 *B* and *C*), producing no or very few pollen grains that could not be stained with I_2_-KI (*SI Appendix*, Fig. S1*D*). Transverse sectioning of Shen95(FA)A showed that the tapetum cells were vacuolated and expanded abnormally compared to those in the Shen95B at stage 9 of anther development. Most microspores of Shen95(FA)A gradually degenerated and disintegrated compared to those in Shen95B at stages 10 to 12 of anther development (*SI Appendix*, Fig. S1*E*).

### The Chimeric Gene *FA182* Causes Male Sterility in CMS-FA.

To identify the gene that causes CMS traits in the CMS-FA line, we sequenced the Shen95(FA)A mitochondrial genome, which is 457,380 bp in total with a 43.83% G+C content (*SI Appendix*, Fig. S2). We compared the mitochondrial genome sequences of four rice varieties, the CMS-FA line Shen95(FA)A, the restorer line 9311, the CMS-RT98 line RT98C, and the maintainer line WA-N (*SI Appendix*, Fig. S3*A*). Two highly diverged regions, which contained four predicted ORFs in each of the regions (*orf56*, *orf205*, *orf126*, and *orf182* in region 1; *orf51*, *orf86*, *orf61*, and *orf55* in region 2), were specifically found in the Shen95(FA)A mitochondrial genome and were subjected to further investigation (*SI Appendix*, Fig. S3*B*). Using BLASTN, *orf56*, *orf205*, and *orf126* were detected in the mitochondrial genome of Nipponbare, a *japonica* rice variety, whereas *orf61* showed an unknown origin. Intriguingly, *orf51* and *orf55* showed high identity (91 to 99%) to mitochondrial genome sequences of the other grasses but not rice, while *orf182* and *orf86* were two chimeric genes generated by the fusion of two or more fragments with different origins. *Orf86* was composed of a segment with unknown origin and a very short fragment having a homologous sequence to mitochondrial sequences of *Zea mays*, *Sorghum bicolor*, and *Eleusine indica*. *Orf182* was formed by merging three components, a 421-bp fragment showing 79% nucleotide identity to a mitochondrial sequence in *Sorghum bicolor*, a 71-bp fragment identical to a mitochondrial sequence in rice, and a 57-bp fragment with unknown origin. Interestingly, *orf182* is identical to the *orf182* gene for CMS in CMS-D1 rice (12); we thus selected *orf182* as the candidate gene for CMS-FA for subsequent investigation.

**Fig. 1.**
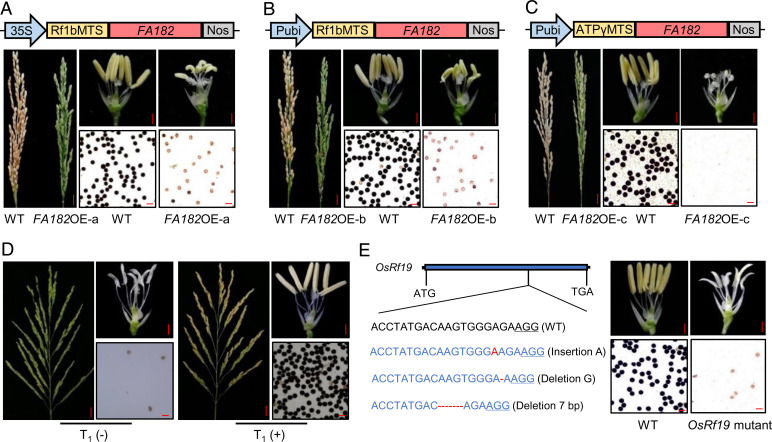
Functional analysis of the CMS gene *FA182* and the restorer gene *OsRf19*. (*A*) Transformation vector 35S-Rf1bMTS-*FA182* and phenotypes of panicles, anthers and pollen grains of the *FA182* overexpression transgenic plants. 35S, the CaMV35S promoter; Rf1bMTS, the mitochondrial transit peptide sequence derived from the Rf1b gene. (*B*) Transformation vector Pubi-Rf1bMTS-*FA182* and phenotypes of panicles, anthers and pollen grains of the *FA182* overexpression transgenic plants. Pubi, the maize ubiquitin promoter. (*C*) Transformation vector Pubi-ATPγMTS-*FA182* and phenotypes of panicles, anthers and pollen grains of the *FA182* overexpression transgenic plants. ATPγMTS, the mitochondrial transit peptide sequence from the ATPγ gene. (*D*) Functional complementation analysis of *OsRf19* (*ORF1*) in rescuing CMS-FA sterility. The panicles, anthers, and 1% I_2_-KI–stained pollen grains for the transgenic T_1_ negative (−) and positive (+) plants carrying *ORF1* are shown. (*E*) Gene editing of *OsRf19* by CRISPR/Cas9 resulting in male sterility. Insertion and deletion in *OsRf19* are indicated. The anthers and 1% I_2_-KI–stained pollen grains for the wild-type (WT) and mutant plants are shown. (*A*–*E*) Scale bars, 2 cm (spikelet); 1 mm (anthers); 50 µm (pollen).

To determine whether *orf182* is the direct cause of male sterility in CMS-FA, three chimeric expression cassettes were constructed with the *orf182* sequence driven by the constitutive promoter CaMV35S or Pubi, and a mitochondrial transit peptide sequence (MTS) from *Rf1b* or *ATPγ* was added to the N terminus of *orf182* to enable its transport from the cytoplasm into mitochondria (Fig. 1*A*). The three plasmids were transformed into the variety Zhonghua11 (*Oryza sativa* ssp. *geng*/*japonica*). A total of 15 independent positive transgenic plants with the 35S-Rf1bMTS-*orf182* vector were obtained, of which nine plants had unstainable pollen grains with no seed setting (Fig. 1*A* and *SI Appendix*, Table S1), six plants had 1 to 50.2% I_2_-KI–stainable pollen grains, and the seed setting rate was 0 to 12.8%. Nine independent positive transgenic plants with the Pubi-Rf1bMTS-*orf182* vector were obtained, of which five plants had unstainable pollen grains and the other four plants had 1 to 53.6% I_2_-KI–stainable pollen grains (Fig. 1*A*). Twenty-one independent positive transgenic plants with the Pubi-ATPγMTS-*orf182* vector were obtained, which had abnormal anthers and unstainable pollen grains (almost no pollen grains), and the seed setting rate was 0. Three male sterile independent T_0_ plants from each transformation construct were randomly chosen to cross with wild-type plants. In the F_1_ segregation populations, the fertile plants without *orf182* and the sterile plants with *orf182* showed a 1:1 segregation ratio (*SI Appendix*, Table S1). These results suggested that *orf182* is the cause of male sterility; we thus named it *FA182*.

We sequenced the RNA transcripts of *FA182* from panicle tissue of Shen95(FA)A using PCR-amplified complementary DNAs (cDNAs). Sequence analysis showed that C-to-U RNA editing occurred at 62 nt and 65 nt of *FA182*. The C to U editing in the 62 nt of *FA182* led to an amino acid change of proline to leucine in the predicted protein, and the editing at 65 nt resulted in a change from serine to phenylalanine. The editing rates were 76.2% at 62 nt and 82.1% at 65 nt of *FA182*.

To determine whether the edited version *FA182UU* is functional in causing male sterility in CMS-FA, an expression cassette Pubi-ATPγMTS-*FA182UU* was constructed with the *FA182UU* sequence driven by the constitutive promoter Pubi and the MTS from *ATPγ* (*SI Appendix*, Fig. S4*A*). The construct was transformed into Zhonghua11. All the transgenic T_0_ plants showed abnormal anthers and no or unstainable pollen grains, the same male sterility phenotype as Shen95(FA)A (*SI Appendix*, Fig. S4*B*). In the F_1_ segregation populations from T_0_ plants crossed with wild-type plants, the fertile plants without *FA182UU* and the sterile plants with *FA182UU* showed a 1:1 segregation ratio (*SI Appendix*, Table S1). Thus, *FA182UU* and *FA182* have the same effect in causing male sterility in rice.

### Map-Based Cloning of *OsRf19*.

We prepared two progeny pools using an F_2_ population from the cross Jinnong2(FA)A × Jinhui3: a fertile pool obtained by mixing the DNA from 10 highly fertile individuals and a sterile pool of 20 completely sterile individuals. RICE6K microarray (38) analysis revealed single nucleotide polymorphisms between the two pools from 17.16 to 19.9 Mb on chromosome 10 (*SI Appendix*, Fig. S5*A*), which coincides with a previous mapping region (36).

We obtained another F_2_ population consisting of 2,096 plants from the same cross. Using markers RM6100 and RM171, we identified 25 recombinants and determined their genotypes at the *OsRf19* locus by progeny testing. This analysis mapped *OsRf19* between the markers Rf1D6 and Rf1D7 (*SI Appendix*, Fig. S5*B*). Subsequently, we constructed a BC_2_F_2_ population consisting of 4,059 plants from the cross of Jinhui3 (with FA cytoplasm) and Huazhan and detected seven recombinants between markers Rf1D6 and Rf1D7. Using information from these recombinants, we delimited *OsRf19* between two markers, Rf1D3 and TMRf1M10, spanning a 16-kb region based on the Nipponbare genome.

We constructed a bacterial artificial chromosome (BAC) library of genomic DNA from Jinhui3, consisting of 41,472 clones with an average DNA insert size of 114 kb. This library was screened with the markers TMRf1M06, Rf1D3, and Rf1D7 using the DNA pooling method. We isolated two BAC clones (71-*N*-20 and 90-J-22) that might cover the corresponding region of the Jinhui3 genome and determined their nucleotide sequences. A 194.7-kb sequence was obtained based on the two BAC clones. The region between markers Rf1D3 and TMRf1M10 in the Jinhui3 genome is 35 kb, 19 kb longer than the corresponding region in the Nipponbare genome.

Four ORFs, *ORF1*, *ORF2*, *ORF3*, and *ORF4*, were predicted in this region (*SI Appendix*, Fig. S5*B*). To identify *OsRf19*, we constructed subclone libraries of the two BAC clones using binary vectors. Three clones (71-*N*-20–06, 71-*N*-20–18, and 90-J-22-09) were identified to contain the genomic sequences, including promoters, ORFs, and 3′ downstream regions of *ORF1*+*ORF2*, *ORF2*+*ORF3*, and *ORF3*+*ORF4*. Four PCR fragments containing the genomic sequences for *ORF1*, *ORF2*, *ORF3*, and *ORF4*, including the promoter ORF and 3′ downstream region, were also individually cloned into binary vectors. These seven constructs were introduced into the CMS-FA line 9311(FA)A (*SI Appendix*, Fig. S6) by *Agrobacterium*-mediated transformation. The positive transgenic T_0_ plants with *ORF1*+*ORF2* (three plants) and *ORF1* (eight plants) showed restored male fertility, producing pollen grains stainable with I_2_-KI. No fertile T_0_ plants were obtained in transgenic plants of the other five constructs. T_1_ seeds from *ORF1*+*ORF2–* and *ORF1*-positive plants were obtained. In three T_1_ families of *ORF1*+*ORF2*, a total of 139 plants with the transgene produced nearly 100% I_2_-KI–stainable pollen grains showing 68.2 to 91.7% seed-setting rates, while 45 plants without the transgene were completely male sterile (*SI Appendix*, Fig. S7). In three T_1_ families of *ORF1*, the 172 transgenic plants with all stained pollen grains showed spikelet fertility of 64.4 to 92.0%, but the 56 plants without the transgene were completely male sterile (Fig. 1*B*). These results indicated that *ORF1* restores the fertility of CMS-FA lines and is the *OsRf19* gene.

We also generated an *OsRf19* knockout mutant using CRISPR/Cas9 in the iso-cytoplasmic restorer line 9311(FA)R, which has *OsRf19* in the 9311 background and CMS-FA cytoplasm. Seventeen independent transgenic T_0_ plants were obtained showing complete sterility (Fig. 1*C*). Three of these T_0_ plants were crossed with 9311 and 9311(FA)R. The F_1_ plants derived from the cross of the T_0_ plants with 9311 showed complete sterility, whereas plants derived from the cross with 9311(FA)R were fertile. Progeny plants resulting from self-fertilization of the fertile plants above showed a 3:1 segregation ratio between the fertile and sterile plants. This result further supported the conclusion that *ORF1* is *OsRf19*.

*OsRf19* encodes a PPR protein, which was predicted to localize to the mitochondria (aias.biol.uoa.gr/PredSL/). To verify this prediction, a transformation construct expressing an OsRF19–green fluorescent protein (GFP) fusion protein was made and introduced into rice protoplasts. Confocal laser-scanning microscopy showed that the green fluorescence of OsRF19-GFP exclusively colocalized with mCherry fluorescence in the mitochondria (*SI Appendix*, Fig. S8).

### OsRF19 Reduces the Cytotoxicity to Bacteria Caused by FA182.

Several CMS-associated genes encode peptides that are deadly to *Escherichia coli* (3, 39). To evaluate whether the expression of FA182 and the edited version FA182UU was also cytotoxic to bacteria, we cloned the coding sequences of *FA182* and *FA182UU* into the first multiple cloning site of the pETDuet1 vector. Expression of either *FA182* or *FA182UU* in *E. coli* induced by Isopropyl-β-D-thiogalactopyranoside (IPTG) hindered *E. coli* growth (*SI Appendix*, Fig. S9*A*).

To investigate whether *OsRf19* mitigates the toxicity caused by *FA182*, we cloned the coding sequence of *OsRf19* into the second multiple cloning site of the pETDuet1 vector, which contained *FA182* in the first multiple cloning site, to coexpress the two genes. The vector containing only the *OsRf19* gene in the second multiple cloning site of pETDuet1 was used as the control; the expression of *OsRf19* did not affect the growth of *E. coli*. Coexpression of the *OsRf19* and *FA182* genes in *E. coli* removed the cytotoxicity to bacteria caused by FA182 as the bacteria continued to grow (*SI Appendix*, Fig. S9*B*).

### OsRF19 Mediates RNA Cleavage of *FA182*.

**Fig. 2.**
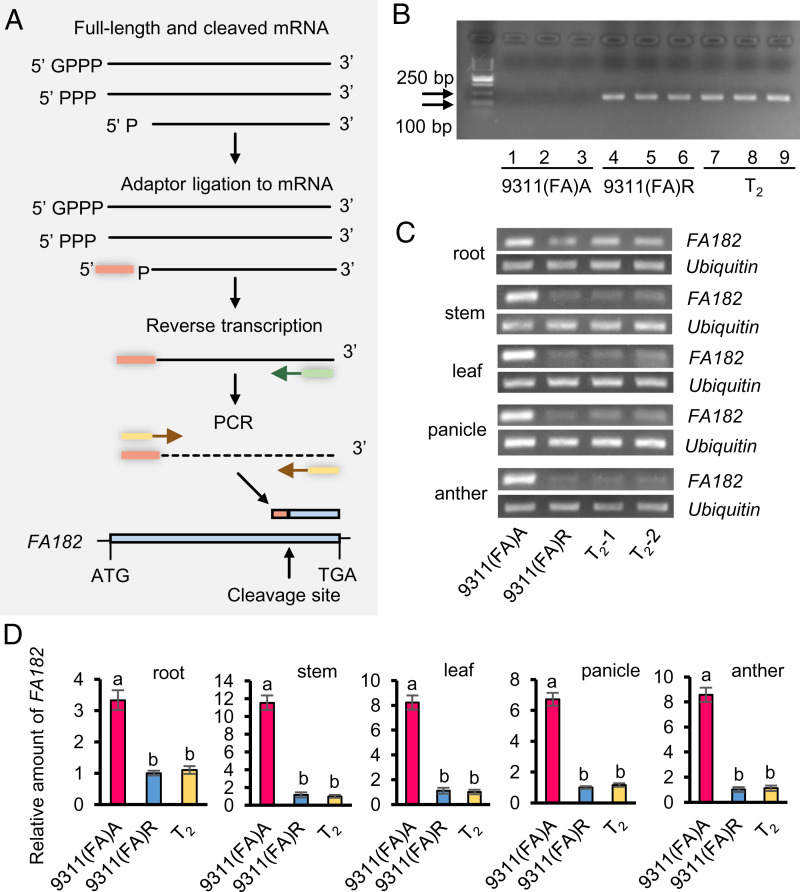
Cleavage analysis of *FA182* transcripts mediated by OsRF19. (*A*) Strategy for ligation-mediated amplification of the 5′ terminus of cleaved *FA182* mRNA for analysis of the cleavage site. Solid line, RNA; dotted line, cDNA; pink box, ligated fragment; green box, reverse transcription primer; and yellow boxes, PCR primers. (*B*) Agarose gel electrophoresis analysis of the PCR products from 9311(FA)A, 9311(FA)R, and *OsRf19* transgenic T_2_ plants. (*C*) RT-PCR assays of *FA182* (26 cycles) in root, stem, leaf, panicle, and anther of 9311(FA)A, 9311(FA)R, and the transgenic T_2_ plants using primer FA182-1F/1R. *Ubiquitin* (28 cycles) was used as the control. (*D*) qRT-PCR analysis of the expression of *FA182* in root, stem, leaf, panicle, and anther of 9311(FA)A, 9311(FA)R, and the transgenic T_2_ plants using primer FA182-1F/1R. Values are presented as the means ± SD (*n* = 3). Different letters denote significant differences determined by Tukey’s tests, *P* < 0.01.

To determine how OsRF19 affects the mRNA of *FA182*, we employed 5′ RNA ligase–mediated rapid amplification of cDNA ends (RLM-RACE) to analyze the cleavage of *FA182* using RNA samples prepared from the panicles of 9311(FA)A, 9311(FA)R, and *OsRf19* transgenic T_2_ plants (Fig. 2*A*). Ligation was carried out by incubating RNA from the samples with a 45-base RNA adapter oligonucleotide mediated by T4 RNA ligase, which would ligate with cleaved RNA but not the CAP-protected uncleaved RNA. A PCR assay was employed to detect cDNA in the samples. Agarose gel electrophoresis detected amplified DNA from 9311(FA)R and T_2_ plants, but no product from 9311(FA)A (Fig. 2*B*). The PCR products from 9311(FA)R and T_2_ plants were cloned for DNA sequencing, which showed that the *FA182* mRNA was cleaved between 414 and 415 nt. Thus, OsRF19 mediates RNA cleavage of *FA182*.

To quantify how much of the *FA182* transcript was reduced by OsRF19, we first analyzed the expression patterns of *OsRf19* and *FA182* in the CMS-FA lines 9311(FA)R and 9311(FA)A, respectively, using qRT-PCR. The transcript level of *OsRf19* was relatively low in the roots, stems, and leaves but high in anther, while *FA182* was high in all the tissues assayed (*SI Appendix*, Fig. S10*A*).

A pair of primers (FA182-1F/1R) flanking both sides of the *FA182* cleavage site was used to compare the transcripts of *FA182* in 9311(FA)A, 9311(FA)R, and *OsRf19* transgenic T_2_ plants by RT-PCR and qRT-PCR. The results showed that the transcript levels were much lower in 9311(FA)R and T_2_ plants than in 9311(FA)A in all tissues, as listed above (Fig. 2 *C* and *D*). Compared with 9311(FA)R, the mRNA level of *FA182* in 9311(FA)A was ∼3.3 times higher in root, 11.5 times higher in stem, 8.2 times higher in leaf, 6.7 times higher in panicle, and 8.5 times higher in anther (Fig. 2*D*).

Another pair of primers (FA182-2F/2R) located upstream of the *FA182* cleavage site was also used to detect the transcript of *FA182* in 9311(FA)A, 9311(FA)R, and *OsRf19* transgenic T_2_ plants. Similar to the above result, the transcript levels of *FA182* were much lower in 9311(FA)R and T_2_ plants than in 9311(FA)A, suggesting that cleaved *FA182* was mostly degraded (*SI Appendix*, Fig. S10*B*). These results confirmed that *OsRf19* affects the transcript level of *FA182*; as much as ∼90% of the *FA182* transcripts were degraded in anthers in the restorer line. The results also suggest that reduction of the *FA182* transcript also occurred in tissues other than the anther.

### Origin of *OsRf19* through Stepwise Duplications in the *Oryza* Genus.

Five PPR genes, *PPR830*, *OsRf19*, *ORF2*, *ORF3*, and *ORF4*, are positionally clustered in a linear order on chromosome 10 in the Jinhui3 genome. (The GenBank accession no. of the Jinhui3 BAC clone sequence containing *OsRf19* is ON855493.) To assess the relationship among members in this cluster, we first compared the coding sequence of *OsRf19* with each of the other four PPRs (*SI Appendix*, Fig. S11*A* and Dataset S1). *PPR830* showed the highest identity in the predicted coding sequence with *OsRf19* (95.1%), followed by *ORF3* (94.6%), *ORF4* (92.6%), and *ORF2* (91.4%). Considering the high similarity in the coding region, we also analyzed the adjacent sequence of *OsRf19* and found that the downstream regions of *OsRf19* and *PPR830* were extremely conserved, with sequence similarity up to 99.7% in the ∼6-kb fragment, whereas the downstream regions of the other three PPRs were more divergent relative to those of *OsRf19*. These data suggested that *OsRf19* was evolutionarily more closely related to *PPR830*. Likewise, high levels of sequence identity were found between *ORF3* and *ORF4* in the upstream region (∼2 kb, 99.0%), coding sequence (2.3 kb, 96.3%), and downstream region (484 bp, 95.4%), suggesting a very close relationship between these two PPRs. *ORF2* encodes a truncated protein as a result of a premature stop codon, and in the coding sequence along with adjacent regions, it showed higher sequence similarity to *ORF3* (95.1%) and *ORF4* (96.5%) than to *PPR830* (92.6%) and *OsRf19* (91.4%).

**Fig. 3.**
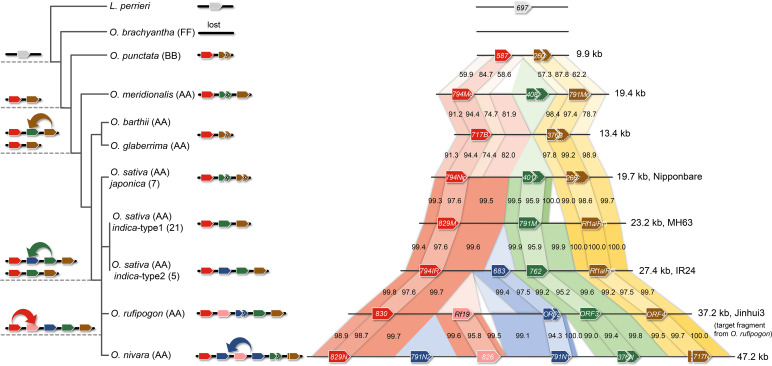
Illustration of the process for origination of *OsRf19*. Comparative analysis of the corresponding *OsRf19* orthologous regions from the *Oryza* genus and *L. perrieri*. The names of the *PPR* genes are labeled in the pentagons. The values indicate the sequence identities of the syntenic regions. The arrows indicate duplication events.

To trace the evolutionary processes stepwise for each of these PPR genes, we performed a comparative sequence analysis of the *OsRf19* orthologous regions from the *Oryza* genus, including *Oryza brachyantha* (FF genome type, accession no. PRJNA70533), *Oryza punctata* (BB, accession number PRJNA13770), *Oryza meridionalis* (AA, accession no. PRJNA48433), *Oryza barthii* (AA, accession no. PRJNA30379), *Oryza glaberrima* (AA, accession no. PRJNA13765), *Oryza rufipogon* (AA), *Oryza nivara* (AA, accession no. PRJNA48107), and *xian*/*indica* and *geng*/*japonica* subspecies of *O. sativa* (AA), using reciprocal-best alignments across the phylogeny (Fig. 3 and *SI Appendix*, Fig. S11*B* and Table S2) (2, 40–42). We also included the closely related species *Leersia perrieri* (accession no. PRJNA163065) from a grass genus as an outgroup in the analysis. A single PPR copy was detected in *L. perrieri* in the syntenic region, suggesting that the origin of this PPR cluster can be traced back to an ancestor predating the occurrence of *L. perrieri*. The copy number of PPRs in this syntenic region differed greatly among the *Oryza* species as a consequence of gene duplications, while the PPR copy in this region of *O. brachyantha* might have been lost since the divergence of this species. (Alternatively, it was lost only in the accession used for genome sequencing, but not the entire species. We assume it is lost in the species in this writing based on the available data.) The ancestral copies of *PPR830* and *ORF4* appeared initially in the most recent common ancestor of the AA and BB lineages and were presumably generated by a local duplication event of a single progenitor gene.

Subsequently, independent duplication events occurred in these two ancestral PPR copies after the split of the AA lineage. The orthologs of *ORF3* might originate from *ORF4* by duplication inferred from their high similarity and adjacent genomic location, and they were preserved in *O. meridionalis* and other Asian species but were not detected in the African species possibly due to limited sample size (Fig. 3). Afterward, duplication of *ORF3* in the most recent common ancestor of Asian rice species gave rise to *ORF2* in *O. rufipogon*, *PPR791N1* and *PPR791N2* in *O. nivara*, and *PPR683* in the *xian*/*indica* subspecies of *O. sativa*. Interestingly, the ortholog of *ORF4* in this lineage gained restoration function to become the restorer gene *OsRf1a*/*OsRf5* for Boro II and Hong-Lian CMS (3, 15).

In the other lineage, *PPR830* gave rise to *OsRf19* through a recent duplication in the wild relatives of Asian cultivated rice, as supported by the observed high sequence similarity and collinearity. Hence, two fertility restorer genes, *OsRf1a*/*OsRf5* and *OsRf19*, were found to originate from a common ancestor predating the split between *L. perrieri*. *OsRf1a*/*OsRf5* represented an older PPR gene arising by an ancient duplication before the split of AA and BB lineages in the *Oryza* genus, while *OsRf19* was a young PPR gene of a recent origination after the divergence of *O. rufipogon* and *O. nivara*. Consequently, *OsRf1a* and *OsRf19* maintained 93.5% sequence identity after experiencing a range of evolutionary changes in their respective lineages, resulting in a distinct fertility restoration spectrum for different types of cytoplasmic male sterility.

We also noticed that the adjacent genomic region surrounding *OsRf19* on chromosome 10 is a hotspot for fertility restorer genes. The restorer line MH63 contains the restorer gene *OsRf1a*/*OsRf5* for Boro II and Hong-Lian cytoplasmic male sterility, the *OsRf1b* for Boro II cytoplasmic male sterility, and two copies of *OsRf4* for WA CMS (*SI Appendix*, Fig. S11*C*). Another three restorer lines, R527, FH838, and R498, also carry *OsRf1a*/*OsRf5* and *OsRf1b* with one copy of *OsRf4*. In addition, 9311 contains two restorer genes, *OsRf1a*/*OsRf5* and *OsRf1b*.

### Application of CMS-FA/*OsRf19* in Hybrid Rice Breeding.

We introduced the *OsRf19* locus from Jinhui3 into six CMS-WA restorer lines, all carrying *OsRf3* and *OsRf4*, HR2168, R498, α7–3, Zhonggeng57, Chenghui727, and Huazhan, through recurrent backcrossing and whole-genome selection using the RICE6K microarray and relevant markers (*SI Appendix*, Fig. S12 *A–C*). Six *OsRf19*-containing lines, HR2168-*OsRf19*, R498-*OsRf19*, α7–3-*OsRf19*, Zhonggeng57-*OsRf19*, Chenghui727-*OsRf19*, and Huazhan-*OsRf19*, were obtained with genetic background recovery rates of 66.2 to 99.9% (*SI Appendix*, Fig. S12*B*). Field evaluation showed that the *OsRf19*-containing lines had essentially the same agronomic performance as their corresponding parents (*SI Appendix*, Table S3).

These *OsRf19* restorer lines plus 9311-*OsRf19*, also named 9311(FA)R, were crossed with the CMS-FA line Shen95(FA)A. For comparison, the original parental lines HR2168, R498, α7–3, Zhonggeng57, Chenghui727, Huazhan, and 9311 were also crossed with the CMS-WA line Shen95(WA)A. Field tests of the F_1_s based on CMS-FA/*OsRf19* showed equivalent or better agronomic performance compared with the corresponding CMS-WA hybrids (Table 1). The yield per plant of the WA-hybrids ranged from 19.3 to 43.0 g, while that of the FA-hybrids ranged from 32.2 to 41.6 g.

**Table 1.** Agronomic performance of test-cross F_1_s in the FA system against WA counterparts

Hybrid	Days to heading	Plant height (cm)	No. of tillers per plant	No. of grains per panicle	Spikelet fertility (%)	1,000-grain weight (g)	Yield per plant (g)
1.1	85.1 ± 0.7	110.2 ± 5.2	10.4 ± 2.0	244.2 ± 33.8	82.9 ± 9.3	20.5 ± 0.8	43.0 ± 10.7
1.2	84.2 ± 0.8	107.2 ± 4.5	10.7 ± 2.7	220.4 ± 44.8	85.8 ± 3.7	20.7 ± 1.0	41.6 ± 12.5
2.1	79.2 ± 0.6	115.1 ± 5.7	9.1 ± 2.9	190.5 ± 20.8	79.3 ± 7.8	24.1 ± 0.9	34.3 ± 16.0
2.2	79.1 ± 0.9	115.5 ± 1.9	8.8 ± 2.4	195.0 ± 21.3	87.9 ± 6.2	24.8 ± 1.0	38.5 ± 15.1
3.1	86.1 ± 0.6	119.7 ± 4.5	8.5 ± 2.3	215.2 ± 36.1	63.0 ± 8.6	25.1 ± 1.1	29.0 ± 10.3
3.2	86.9 ± 0.6	115.3 ± 4.1	7.9 ± 1.5	211.6 ± 39.6	74.2 ± 7.0	26.0 ± 0.7	32.2 ± 9.9
4.1	81.6 ± 0.5	113.9 ± 3.7	10.1 ± 2.8	194.7 ± 36.5	77.1 ± 7.3	22.5 ± 0.7	35.8 ± 17.0
4.2	82.7 ± 0.7	116.1 ± 3.2	8.9 ± 2.5	203.4 ± 31.1	80.2 ± 9.5	23.4 ± 1.3	35.0 ± 13.8
5.1	83.4 ± 0.7	109.5 ± 4.2	9.2 ± 2.7	190.4 ± 23.0	87.4 ± 5.5	25.2 ± 0.7	39.3 ± 15.1
5.2	83.6 ± 0.5	106.7 ± 4.3	9.9 ± 3.1	181.3 ± 25.8	89.8 ± 3.3	25.2 ± 0.6	41.0 ± 14.3
6.1	81.4 ± 0.5	106.6 ± 4.3	9.1 ± 2.8	260.4 ± 38.3	55.5 ± 9.2	22.1 ± 0.7	29.1 ± 11.6
6.2	80.6 ± 0.5	107.0 ± 6.3	8.8 ± 3.3	242.7 ± 29.6	81.4 ± 7.1*	22.4 ± 1.2	38.3 ± 14.1*
7.1	90.3 ± 0.7	113.9 ± 4.9	10.2 ± 2.6	241.4 ± 35.5	31.1 ± 12.2	24.7 ± 1.3	19.3 ± 9.1
7.2	89.4 ± 0.5	118.4 ± 5.0	9.2 ± 2.4	233.6 ± 39.6	75.4 ± 6.4*	24.5 ± 0.9	39.8 ± 13.3*

1.1 Shen95(WA)A/Huazhan, 1.2 Shen95(FA)A/Huazhan-*OsRf19*; 2.1 Shen95(WA)A/HR2168, 2.2 Shen95(FA)A/HR2168-*OsRf19*; 3.1 Shen95(WA)A/R498, 3.2 Shen95(FA)A/R498-*OsRf19*; 4.1 Shen95(WA)A/α7–3, 4.2 Shen95(FA)A/α7–3-*OsRf19*; 5.1 Shen95(WA)A/Chenghui727, 5.2 Shen95(FA)A/Chenghui727-*OsRf19*; 6.1 Shen95(WA)A/Zhonggeng57, 6.2 Shen95(FA)A/Zhonggeng57-*OsRf19*; 7.1 Shen95(WA)A/9311, 7.2 Shen95(FA)A/9311-*OsRf19* (9311 is not a restorer of CMS-WA). Values are presented as the means ± SD.

^*^Significant difference from the performance of the F_1_s (FA) when compared with F_1_s (WA) at *P* < 0.01.

These *OsRf19*-restorer lines were also crossed with the commercial CMS-FA line Jinnong3A. The hybrids showed excellent performance compared with the check Fengliangyou4, a popular high-yielding two-line hybrid (*SI Appendix*, Table S4). The grain yield per plant of hybrids from the cross of Jinnong3(FA)A with these restorer lines ranged from 32.4 to 38.6 g compared to 32.0 g for Fengliangyou4. In particular, in the field plot trial, grain yields of the three FA-combinations, Shen95(FA)A/Huazhan-*OsRf19*, Jinnong3(FA)A/Yuehui94-*OsRf19*, and Jinnong3(FA)A/Jinhui3-*OsRf19*, were 10.9, 11.8, and 12.3 ton/ha, respectively, and the yield of the check Fengliangyou4 was 10.8 ton/ha (*SI Appendix*, Table S5). These results demonstrate that the CMS-FA/*OsRf19* system holds great promise for hybrid rice breeding.

## Discussion

**Fig. 4.**
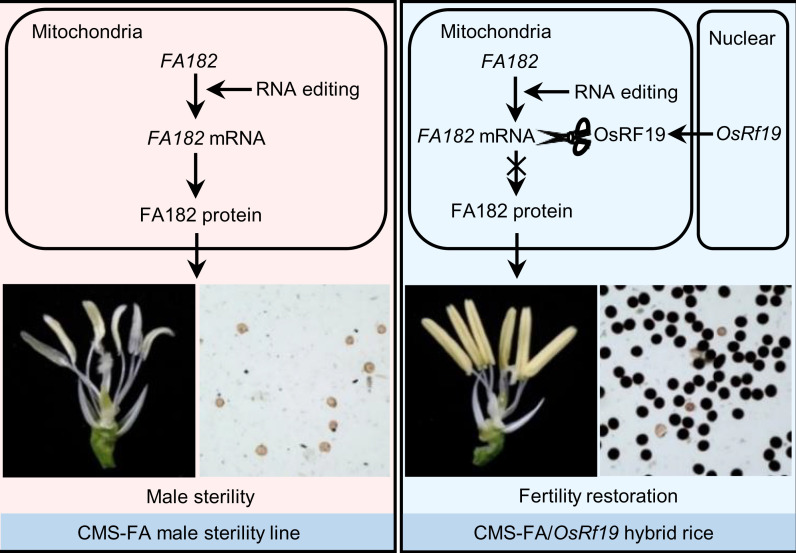
The proposed model illustrating the mechanism of CMS and fertility restoration in CMS-FA rice. Without the restorer protein, the mitochondria-encoded gene *FA182* is transcribed, edited, and translated into FA182 protein, which causes male sterility in the CMS-FA line (*Left*). In the presence of the nuclear-encoded OsRF19 protein, the *FA182* transcript is cleaved and the abundance is greatly reduced, leading to fertile pollen grains in CMS-FA/*OsRf19* hybrid rice (*Right*).

Based on our results, we proposed a model to illustrate the mechanism of CMS and fertility restoration in CMS-FA rice (Fig. 4). Without the restorer protein, the mitochondria-encoded gene *FA182* is transcribed, and the transcript, with or without editing, is translated into the FA182 protein, which causes male sterility in the CMS-FA line (Fig. 4). In the presence of the nuclear-encoded OsRF19 protein, the *FA182* transcript is cleaved, to avoid causing male sterility in CMS-FA/*OsRf19* hybrid rice.

RNA editing in plant mitochondria or chloroplasts is a posttranscriptional process that directs C to U changes in RNA (43, 44). RNA editing plays many important roles in gene expression by generating start codons at ACG sites, correcting codons to encode conserved amino acids, and generating required stop codons. Such editing may change the biochemical nature of the resulting proteins. An analysis of RNA editing in *Arabidopsis* mitochondria showed that 35% of the modifications of the codons altered the amino acids from hydrophilic to hydrophobic, while 35% were hydrophobic to hydrophobic (45). Thus, the general tendency of the effect of RNA editing in *Arabidopsis* mitochondria is to increase the proportion of hydrophobic amino acid codons. In addition, RNA editing may increase the stability and thus the quantity of the protein. For example, in maize, six C to U editing sites have been identified in the transcripts of the ribosomal protein S13 gene (*rps13*) in the mitochondria (46). Sequence analysis demonstrated that 73% of the transcripts of S13 were edited at all six sites and only 3% were completely unedited. Immunological analyses showed that only polypeptides from the edited RNA are detected and accumulate, whereas polypeptides from unedited RNA do not accumulate in maize mitochondria. This study indicates that translational products from unedited RNAs may be unstable and consequently fail to accumulate (46). In our study, the C to U editing in the 62 nt of *FA182* led to an amino acid change of proline (hydrophobic) to leucine (hydrophobic) in the predicted protein, and the editing at 65 nt resulted in a change from serine (hydrophilic) to phenylalanine (hydrophobic). Therefore, RNA editing may have changed the stability and biochemical nature of the FA182 protein.

Numerous CMS germplasms have been found in various plant species, which in general can be characterized as two classes, gametophytic male sterility with pollen fertility specified by pollen genotype and sporophytic male sterility with pollen fertility decided by the maternal plant. Experience with hybrid rice breeding in the past decades clearly indicates that the gametophytic male sterility systems may suffer two disadvantages: sterility fluctuation of the male sterile lines to occasionally produce small amounts of fertile pollen and suboptimal seed setting of the F_1_ hybrids due to inadequate pollen fertility resulting in yield loss. This explains why the sporophytic CMS-WA/*Rfs* system is still dominant in hybrid rice breeding both in China and other countries. However, obvious difficulties have been encountered with the WA system in hybrid rice breeding. First, the need for simultaneous transfer of two restorer genes causes difficulties in the background improvement of the restorer lines due to linkage drag at two loci and also for phenotyping fertility of the two-locus genotypes. Second, such two-locus segregation also causes complications for breeding of maintainer lines. These difficulties have certainly hindered the development of hybrid rice breeding.

The CMS-FA/*OsRf19* reported here may provide a promising alternative to improve the situation. We showed that the male sterile lines bred using this system produce no pollen, or at least no stainable pollen, thus exhibiting complete male sterility and that a single restorer gene adequately restores male fertility of the hybrids with no negative effects on hybrid performance compared to WA counterparts. We also showed that this single-gene inheritance greatly eases the breeding process of both maintainer and restorer lines. Such progress has demonstrated a perspective that tremendously accelerates the development of hybrid rice breeding, especially when equipped with genomic selection technologies, although its usefulness remains to be tested by large-scale utilization in breeding programs.

A major remaining question is how the PPR protein OsRF19 mediates the cleavage of the *FA182* transcripts to restore pollen fertility. Although we found that expression of *FA182* in *E. coli* hindered cell growth and coexpression of *OsRf19* and *FA182* removed the cytotoxicity, we do not know how *OsRf19* functions to remove the deadly effect of *FA182* in bacterial cells. However, it can be reasoned that the action by which *OsRf19* reduces the toxicity of *FA182* in bacteria is likely different from its function in restoring male fertility in the rice plant. We attempted to assay whether OsRF19 protein binds to the *FA182* transcripts without success. An alternative possibility is that OsRF19 may act on the *FA182* transcript with the participation of other proteins, as in the Hong-Lian CMS line in which OsRF5 physically interacts with GRP162, which has an RNA recognition motif to bind *atp6-orfH79* (17). Likewise, OsRF6 does not directly bind to the *atp6-orfH79* transcript but physically interacts with OsHXK6 and promotes the processing of *atp6-orfH79* (18). Further investigation is needed to answer this question.

## Materials and Methods

Detailed descriptions for characterization of the phenotypes of Shen95(FA)A, mitochondrial genomic sequencing and comparative analysis, vector construction and transformation of *FA182*, RNA editing analysis, mapping of *OsRf19*, construction of a BAC library and two subclone libraries, transformation materials and vector construction for *OsRf19* candidate genes, subcellular localization analysis of OsRF19, expression of *FA182* and *OsRf19* in *E. coli*, RLM-RACE assay, expression analysis, genomic variation analysis of the *OsRf19* locus, and breeding application and trait measurement are provided in *SI Appendix*.

## Supplementary Material

Supplementary File

## Data Availability

All study data are included in the article and/or supporting information.
