# Supplementary material for: Fujian cytoplasmic male sterility and the fertility restorer gene OsRf19 provide a promising breeding system for hybrid rice
Source: Proc Natl Acad Sci U S A. 2022 Aug 15;119(34):e2208759119. doi: 10.1073/pnas.2208759119 (PMC9407659; doi:10.1073/pnas.2208759119)
Supplement: Supplementary File [file pnas.2208759119.sapp.pdf]

Supplementary Information for

Fujian cytoplasmic male sterility and the fertility restorer gene *OsRf19* provide a promising breeding system for hybrid rice

Haichao Jiang, Qing Lu, Shuqing Qiu, Huihui Yu, Zhengji Wang, Zhichao Yu, Yunrui Lu, Lei Wang, Fan Xia, Yuying Wu, Fan Li, Qinglu Zhang, Gang Liu, Dingding Song, Chonglie Ma, Qi Ding, Xiaobo Zhang, Lin Zhang, Xuetang Zhang, Xu Li, Jianwei Zhang, Jinghua Xiao, Xianghua Li, Naiyuan Wang, Yidan Ouyang, Fasong Zhou, Qifa Zhang

Correspondence to:
Yidan Ouyang (diana1983941@mail.hzau.edu.cn);
Fasong Zhou (zhoufasong@greenfafa.com);
Qifa Zhang (qifazh@mail.hzau.edu.cn).

This file includes:

Supplementary Materials and Methods

Figs. S1 to S12

Tables S1 to S6

Dataset S1

References for SI reference citations

Supplementary Materials and Methods

Characterization of the phenotypes of Shen95(FA)A

Shen95(FA)A is a male sterile line of CMS-FA, which was obtained by crossing Shen95B with Jinnong2(FA)A and backcrossing with Shen95B seven times. Shen95B is the maintainer line of the male sterile line Shen95(WA)A of CMS-WA. To observe callose, the anthers of the Shen95(FA)A and Shen95B plants were dissected and vacuum infiltrated with 4% (v/v) paraformaldehyde in phosphate-buffered saline (pH 7.0) for 30 min, renewed with fresh paraformaldehyde solution and incubated overnight at 4°C. Then, the fixed samples were dehydrated in a graded ethanol series and embedded in paraffin. Subsequently, the materials were cut into 3-μm-thick sections using a microtome and stained with toluidine blue to stain the callose walls. Sections were viewed and photographed with an Olympus BX51 microscope.

Mitochondrial genomic sequencing and comparative analysis

Approximately 5 g of fresh leaves of Shen95(FA)A seedlings grown in the dark was harvested and mitochondrial DNA was isolated using an improved extraction method (1). After DNA isolation, 1 μg of purified DNA was fragmented to construct short-insert libraries (insert size ~ 430 bp) according to the manufacturer's instructions (Illumina) and then sequenced on an Illumina HiSeq 4000. High-molecular-weight DNA was purified and used for PacBio library prep and then sequenced on a Sequel Sequencer. The mitochondrial genome was constructed and assembled with the PacBio Sequel data and the Illumina Hiseq data using SPAdes v3.10.1 (2). The mitochondrial genes were annotated using homology alignments and *de novo* prediction. The circular Shen95(FA)A mitochondrial genome map was drawn using Organellar Genome DRAW v1.2. The mitochondrial genome of Shen95(FA)A was uploaded to NCBI (GenBank accession number ON854123).

The mitochondrial genome sequence of Shen95(FA)A was aligned to those of 9311, RT98C and WA-N using Mummer (v 4.0). The raw alignments were further filtered using delta-filtre, and the structural variations were detected using SyRI (3). The circos plot was generated by shinyCircos (4). The mitochondrial genome accession numbers

were DQ167399 for 9311, AP012527 for RT98C, and JF281153 for WA-N.

Vector construction and transformation of *FA182*

The mitochondrial transit peptide sequence of the rice *OsRf1b* restore gene (5) and *ATPγ* gene (6) were fused to the N-terminus of *FA182* (also as *orf182* for ease of presentation depending on the context). Then, these DNA fragments were inserted into the binary vectors pCAMBIA1301S and pCAMBIA1301U, which had been modified to contain the constitutive promoters CaMV35S and Pubi, respectively, and the resulting expression cassettes were CaMV35S::Rf1bMTS-*FA182*::Nos, Pubi::Rf1bMTS-*FA182*::Nos, Pubi::ATPγMTS-*FA182*::Nos. The mitochondrial transit peptide sequence of *ATPγ* was fused to the N-terminus of *FA182UU*. Then, the DNA fragment was inserted into the binary vector pCAMBIA1301U, and the resulting expression cassette Pubi::ATPγMTS-*FA182UU*::Nos. *Agrobacterium tumefaciens* EHA-105 was used to transform the *geng*/*japonica* variety Zhonghua11.

RNA editing analysis

The male sterile line Shen95(FA)A was used in the RNA editing analysis. RNA was isolated from panicle tissue using TRIzol Reagent. Fragments of *FA182* were amplified by reverse transcription RT-PCR using cDNA as the template. The RT-PCR products were directly sequenced at Sangon Biotech (Shanghai) Co., Ltd. The sequences of all primers are listed in Table S6.

Mapping of *OsRf19*

The CMS-FA male sterile line Jinnong2(FA)A and restorer line Jinhui3, the parents of a commercial hybrid Jinnong2You3 (7), were used to develop the F_2 mapping population. For bulked segregant analysis, the fertile pool and sterile pool were derived from 10 extremely fertile individuals and 20 extremely sterile individuals, respectively, selected from the F_2 population. A RICE6K microarray was used to genotype the DNA pools (8).

In total, 2,096 individuals of the F_2 population from Jinnong2You3 were used for

initial mapping of *OsRf19*. We developed another mapping population of BC_2F_2 consisting of 4,059 individuals from a cross of Huazhan, an elite *xian*/*indica* rice in China, with Jinhui3, which was backcrossed twice with Huazhan. SSR and SNP markers were used for fine mapping of *OsRf19* (Table S6).

Construction of a BAC library and two subclone libraries

To construct a bacterial artificial chromosome (BAC) library, the genomic DNA of Jinhui3 was partially digested with *HindIII*, and the recovered fragments were inserted into the vector pIndigoBAC536-S (9). Sequencing of the two BAC clones, 71-N-20 and 90-J-22, was performed using Illumina HiSeq 4000 and PacBio Sequel Sequencer at Frasergen (Wuhan) Co., Ltd.

To construct subclone libraries, the two BAC clones were digested with *Sau3AI*, and fragments were inserted into the vector BIBAC-S (9).

Transformation materials and vector construction for *OsRf19* candidate genes

9311, an elite rice variety, was crossed with Jinnong2(FA)A, and then the progeny was backcrossed with 9311 seven times, resulting in 9311(FA)A. RICE6K microarray analysis showed that 9311(FA)A and 9311 have almost identical nuclear genomes (Fig. S6). 9311(FA)R was derived from a cross between 9311 and wild rice (having *OsRf19* and FA-cytoplasm) followed by backcrossing with 9311 nine times. RICE6K microarray analysis showed that 9311(FA)R contained a fragment from wild rice on chromosome 10 (Fig. S6).

The full-length coding sequences, promoters, and 3' downstream regions of the candidate genes *ORF1*, *ORF2*, *ORF3* and *ORF4* were cloned into the binary vector pCAMBIA1300. Three constructs each containing two genes and four constructs each containing one gene were transformed into 9311(FA)A by *Agrobacterium*-mediated transformation.

To generate *OsRf19* knockout lines, the CRISPR/Cas9 genome editing method was used. A 19-bp coding sequence (5'-ACCTATGACAAGTGGGAGA-3') of *OsRf19* was cloned into a sgRNA-Cas9 expression vector (10) and transformed into 9311(FA)R. A

primer pair surrounding the target region of *OsRf19* was used to amplify the DNA of the transgenic plants, and the derived PCR products were sequenced to determine the mutation site of *OsRf19* (Table S6).

Subcellular localization analysis of OsRF19

The coding region of *OsRf19* without the stop codon was amplified and cloned into the pBI221-GFP vector, which had been modified in the multiple cloning site. CD3-mCherry (CD3-991) was used as the mitochondrial location marker (11). Rice protoplasts were isolated from 12-day-old seedlings (variety 9311). Rice protoplast preparation and the transient expression assay were performed as previously described (12). The green fluorescent protein (GFP) and the mCherry fluorescence protein were observed and photographed using a confocal microscope (FV 1200; Olympus, Tokyo, Japan).

Expression of *FA182* and *OsRf19* in *E. coli*

The pETDuet1 vector contains two multiple cloning sites that can express one gene alone or two genes simultaneously in *E. coli*. The pETDuet1 vector was digested with *BamHI* and *SalI* in the first multiple cloning site and digested with *BglII* and *KpnI* in the second multiple cloning site. *FA182* and *OsRf19* were amplified from Shen95(FA)A rice and 71-N-20-06 subclone, respectively, via PCR using the primers in Table S6. Then, *FA182* was ligated with the first multiple cloning site, and *OsRf19* was ligated with the second multiple cloning site using a DNA assembly method (Assembly mix from New England Biolabs). The plasmids pETDuet1-*FA182*, pETDuet1-*FA182+OsRf19*, and pETDuet1-*OsRf19* and the pETDuet1 empty vector were transformed into BL21 (DE3) strains. Protein expression in *E. coli* DE3 cells was induced via the addition of 1 mM IPTG.

RLM-RACE assay

The procedure of the assay is illustrated in Fig. 2A. Total RNA was isolated with TRIzol reagent (Invitrogen). The RNA from 9311(FA)A, 9311(FA)R and *OsRf19* transgenic T_2

plants was used as a substrate for RLM-RACE using the components of the First Choice RLM-RACE kit (13, 14). RNA from the samples was not treated with calf intestine alkaline phosphatase and tobacco acid pyro-phosphatase to retain the cap structure or triphosphate of the full-length mRNA at the 5’ end and was thus unable to be ligated to an adaptor, while the cleaved transcript with an exposed 5’-monophosphate can be ligated to a RNA adapter of oligonucleotides using T4 RNA ligase. In the ligation step, the 5’ adaptor (45 base) and the RNA were incubated at 37°C for 1 h. The cDNA was made using M-MLV reverse transcriptase with random decamers primer, according to the manufacturer’s instructions. The resulting cDNA was amplified using the 5’ RACE outer primer and gene-specific primer 1. The DNA product from the PCR was further amplified using the 5’ RACE inner primer and the gene-specific primer 2. The products were run on a 1.0% agarose gel, stained with goldview and visualized under UV light. The product from the second PCR was cloned into pEASY-T3 vectors (TransGen Biotech, Beijing) and then sequenced. The primers used are listed in Table S6.

Expression analysis

Total RNA was isolated with TRIzol reagent (Invitrogen). cDNA was obtained through reverse transcription using a HiScript III 1st Strand cDNA Synthesis kit (Vazyme, China) according to the manufacturer’s instructions. Real-time quantitative PCR (qRT-PCR) was performed using the SYBR Green I Master PCR kit on the ABI Vii7. For qRT-PCR analysis of *OsRf19* and *FA182*, RNA samples were prepared from root, stem, leaf, panicle and anther of 9311(FA)A, 9311(FA)R and *OsRf19* transgenic T_2 plants. Root, stem, leaf, and panicle tissues were collected at the meiosis stage, and anthers were collected at stage 11 of panicle development. Each experiment was biologically repeated three times, with three replicates each. The rice *ubiquitin* gene was used as an internal control for normalization. The relative expression levels were measured using the $2^{-\Delta\Delta Ct}$ analysis method (15), and the results are presented as the means ± SD. For RT-PCR analysis of *FA182* and *ubiquitin*, the PCR program was set for 26 and 28 cycles, respectively. All primers used for gene expression analysis are listed in Table S6.

Genomic variation analysis of the *OsRf19* locus

Sequence analysis of the *OsRf19* orthologous regions from the *Oryza* genus, including *O. brachyantha* (FF genome type), *O. punctata* (BB), *O. meridionalis* (AA), *O. barthii* (AA), *O. glaberrima* (AA), *O. rufipogon* (AA), *O. nivara* (AA), *indica* and *japonica* subspecies of *O. sativa* (AA), as well as the closely related outgroup species *Leersia perrieri* from a grass genus (16–19), were performed using reciprocal-best alignments across the phylogeny. The nucleotide sequences were compared using Basic Local Alignment Search Tool (BLAST+/2.9.0). Syntenic relationships between orthologous sequences in *L. perrieri*, *O. brachyantha*, *O. punctata* and *O. sativa* (MH63) are connected using gray lines with identity higher than 80%. Genes were predicted in ORF finder (https://www.ncbi.nlm.nih.gov/orffinder/), and gene structure was predicted in PROSITE (https://prosite.expasy.org/).

The GenBank BioProject codes for *Oryza species* used in this study are as follows: *O. nivara* (PRJNA48107), *O. barthii* (PRJNA30379), *O. glaberrima* (PRJNA13765), *O. meridionalis* (PRJNA48433), *O. punctata* (PRJNA13770), *O. brachyantha* (PRJNA70533) and *L. perrieri* (PRJNA163065). The GenBank accession numbers of the *Rf1a* locus sequence in IR24 is AB110443. The GenBank accession number of the Jinhui3 BAC clone sequence containing *OsRf19* is ON855493. All PPR gene sequences and accession numbers are presented in Dataset S1.

Breeding application and trait measurement

Six commercial restorer lines of the CMS-WA system, HR2168, R498, α7-3, Zhonggeng57, Chenghui727 and Huazhan were used as recurrent parents for developing restorer lines using Jinhui3 as the donor of *OsRf19*. The breeding process involved four backcrosses and two generations of selfing (Fig. S12*A*). In backcrossing generations from BC_1F_1 to BC_4F_1, molecular markers were used to assist in selecting the individual with *OsRf19*. In BC_2F_1, BC_3F_1 and BC_4F_1, the heterozygous plants were subjected to background selection using the whole-genome array RICE6K. The BC_4F_2 and BC_4F_3 plants were obtained from an individual in BC_4F_1 with the highest genome recovery rate of the recurrent parent. Ultimately, six *OsRf19*-containing lines, HR2168-

OsRf19, R498-*OsRf19*, α7-3-*OsRf19*, Zhonggeng57-*OsRf19*, Chenghui727-*OsRf19* and Huazhan-*OsRf19*, were obtained. Then, the new restorer lines and original parental lines were planted in the field with a randomized complete block design with three replications in Hainan in 2019 for agronomic evaluation. Eight individuals in the middle of the second row in each plot were taken for measuring agronomic traits, including number of days to heading, plant height, number of tillers per plant, number of grains per panicle, spikelet fertility, weight of 1000-grains, and yield per plant.

These six new restorer lines and 9311-*OsRf19*, also named 9311(FA)R were crossed with the CMS-FA line Shen95(FA)A. The corresponding WA parents, HR2168, R498, α7-3, Zhonggeng57, Chenghui727, Huazhan and 9311, were crossed with the CMS-WA line Shen95(WA)A. Shen95(WA)A and Huazhan were the parents of the elite hybrid Shen95youhuazhan. Additionally, the *OsRf19*-containing lines HR2168-*OsRf19*, R498-*OsRf19*, α7-3-*OsRf19*, Zhonggeng57-*OsRf19*, Chenhui727-*OsRf19*, Huazhan-*OsRf19*, 9311-*OsRf19*, Yuehui94-*OsRf19* and Jinhui3 were crossed with the commercial CMS-FA line Jinnong3(FA)A. The F_1s were planted in the field with a randomized complete block design with three replications in Wuhan in the summer of 2020 for agronomic evaluation using the elite hybrid rice Fengliangyou4 as a check. Each plot consisted of three rows with 10 plants per row at a planting density of 16.6 cm between plants and 20.0 cm between rows. Eight individuals in the middle of the second row in each plot were taken for measuring agronomic traits, including number of days to heading, plant height, number of tillers per plant, number of grains per panicle, spikelet fertility, weight of 1000-grains, and yield per plant.

Three hybrids, Shen95(FA)A/Huazhan-*OsRf19*, Jinnong3(FA)A/Yuehui94-*OsRf19* and Jinnong3(FA)A/Jinhui3, were planted in plots of 200 plants each in Wuhan in the summer of 2020, with Fengliangyou4 as the check. Grain yield was measured for each plot.

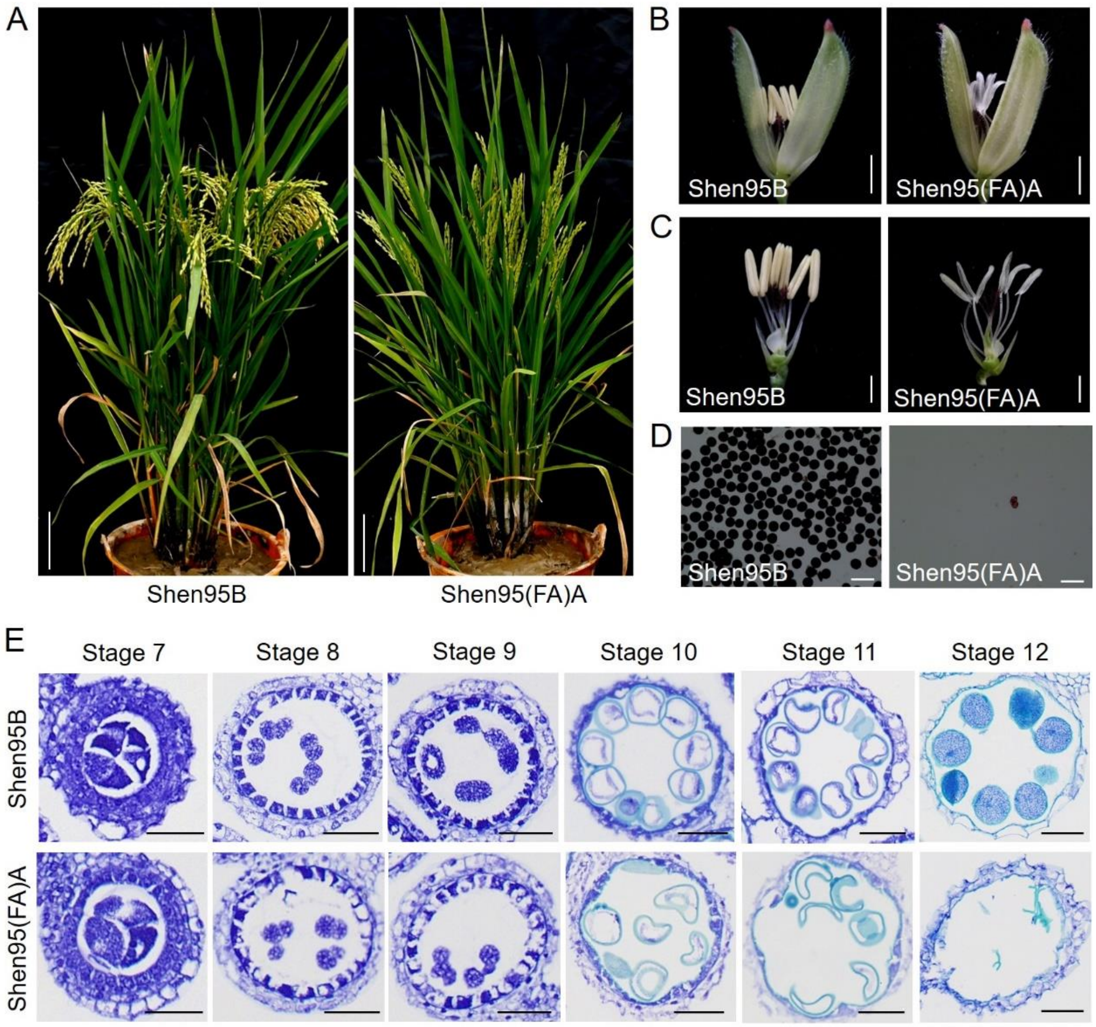

Fig. S1. Phenotypes of Shen95B and Shen95(FA)A. (*A*) Whole plants of Shen95B and Shen95(FA)A at the maturation stage. Scale bars, 10 cm. (*B*) The flowers of Shen95B and Shen95(FA)A. Scale bars, 2 mm. (*C*) The mature anther phenotypes of Shen95B and Shen95(FA)A. Scale bar, 1 mm. (*D*) Pollen grains of Shen95B and Shen95(FA)A stained with 1% I_2-KI. Scale bars, 50 μm. (*E*) Section observation of Shen95B and Shen95(FA)A anthers during stages 7 to 12 of anther development. Scale bars, 20 μm.

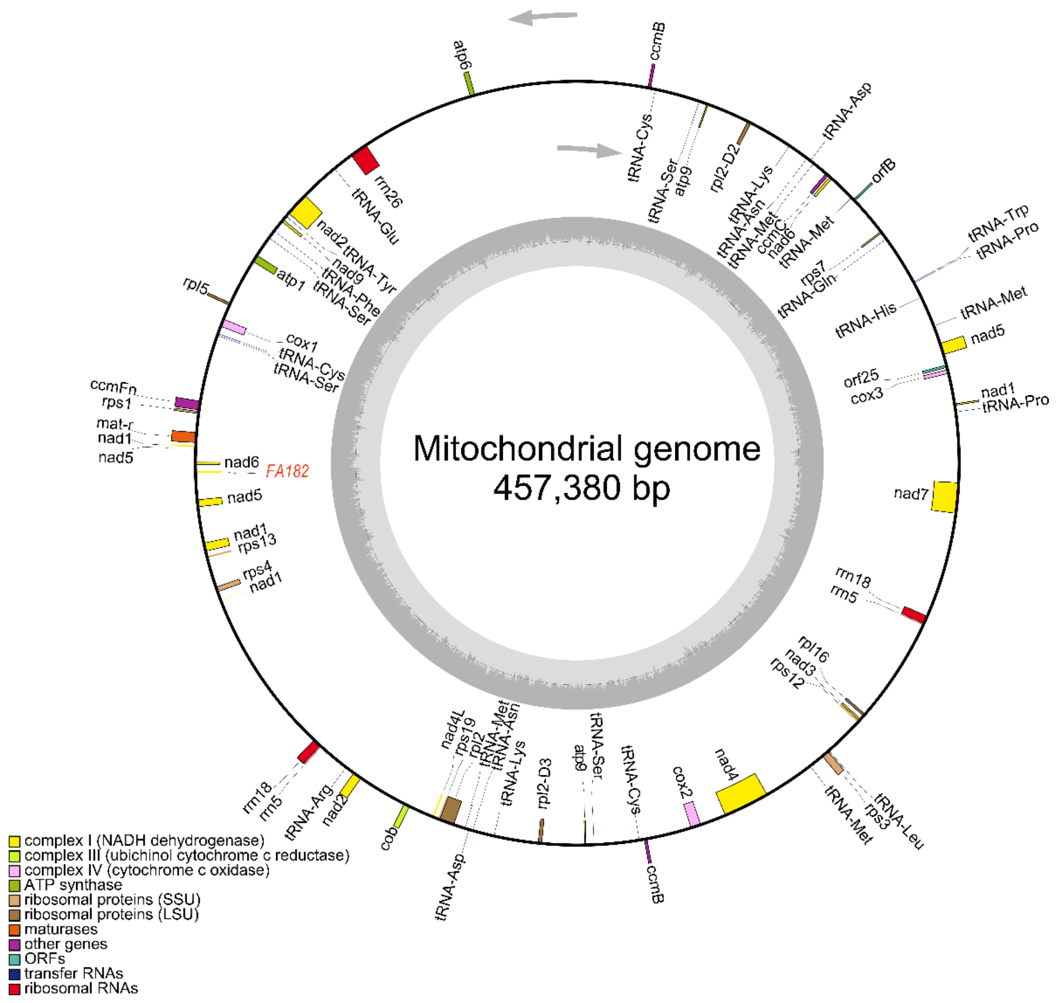

Fig. S2. The mitochondrial genome of the Shen95(FA)A as drawn using circular-mapping. The features of the transcriptionally clockwise and anti-clockwise strands are indicated inside and outside of the circle, respectively.

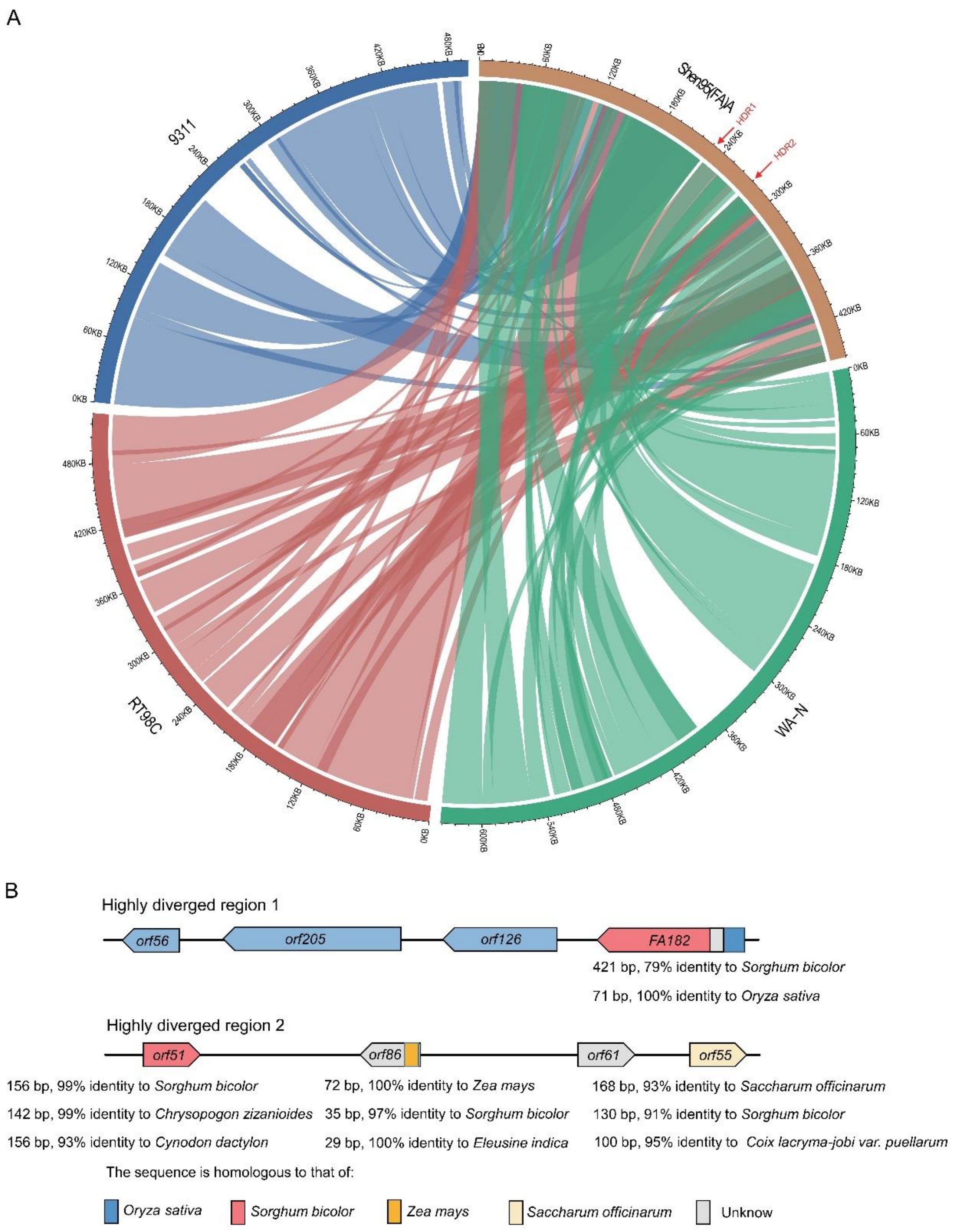

Fig. S3. Comparative sequence analysis of the mitochondrial genomes. (*A*) Synteny of the mitochondrial genomes in four rice varieties, CMS-FA line Shen95(FA)A, 9311, CMS-RT98 line RT98C and a maintainer line WA-N. Lines connect homologous sequences. HDR, highly diverged region. (*B*) ORFs predicted in the two highly diverged regions are indicated by pentagons.

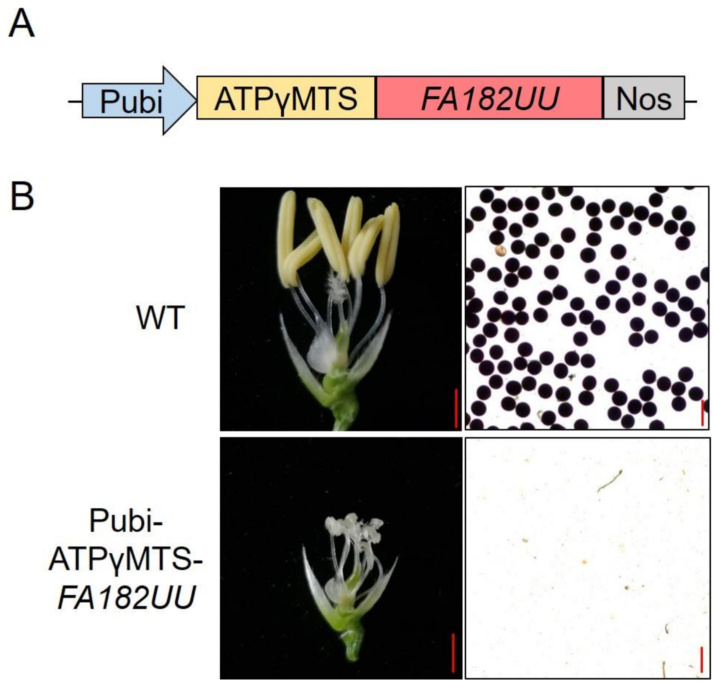

Fig. S4. Confirmation of the function of *FA182UU*. (*A*) Structure of the binary vector Pubi-ATP*γ*MTS-*FA182UU* used for transformation of *FA182UU*. Pubi, the maize ubiquitin promoter. ATP*γ*MTS, the mitochondrial transit peptide sequence derived from the ATP*γ* gene. (*B*) Anther and pollen grains of the wild type (WT) rice and transgenic plants with the Pubi-ATP*γ*MTS-*orf182UU* vector. Pollen grains are stained with 1% I_2-KI. Scale bars, 1 mm (anthers); 50 µm (pollen).

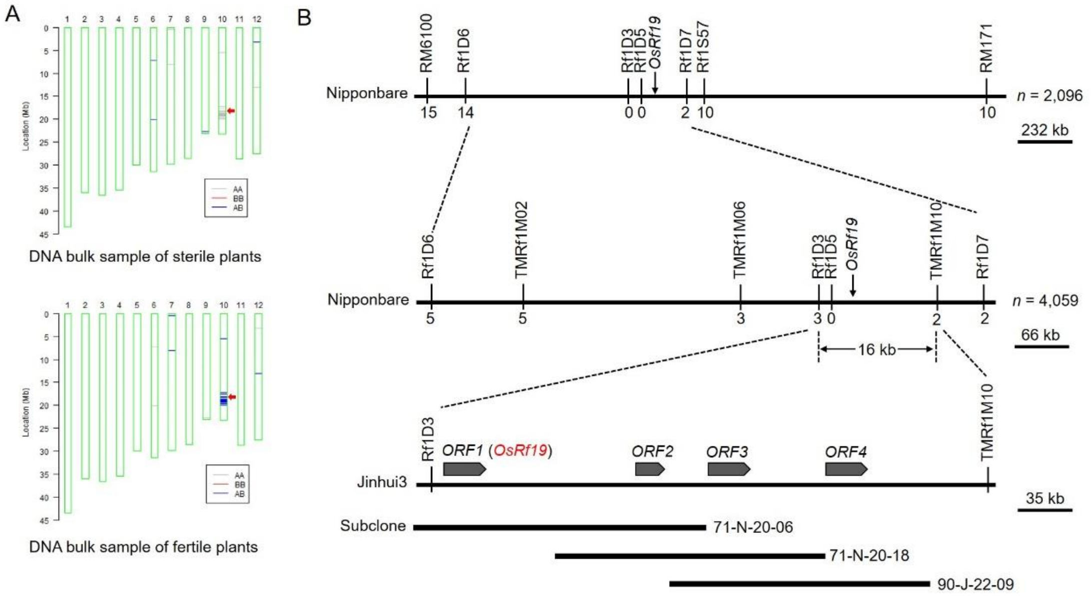

Fig. S5. Mapping the restorer gene *OsRf19*. (*A*) Diagram of genotyping bulked DNA samples from 20 sterile plants and 10 fertile plants using the RICE6K array. The short black and blue lines on the chromosomes represent the single nucleotide polymorphism (SNP) sites between the parents. The red arrow indicates the position of *OsRf19*. (*B*) Fine mapping of *OsRf19* using two mapping populations with 2,096 and 4,059 plants. The numbers underneath the molecular markers indicate the number of recombinants. Four ORFs were predicted based on the Jinhui3 genome. Three subclones were obtained containing the genomic sequences of *ORF1+ORF2*, *ORF2+ORF3* and *ORF3+ORF4*.

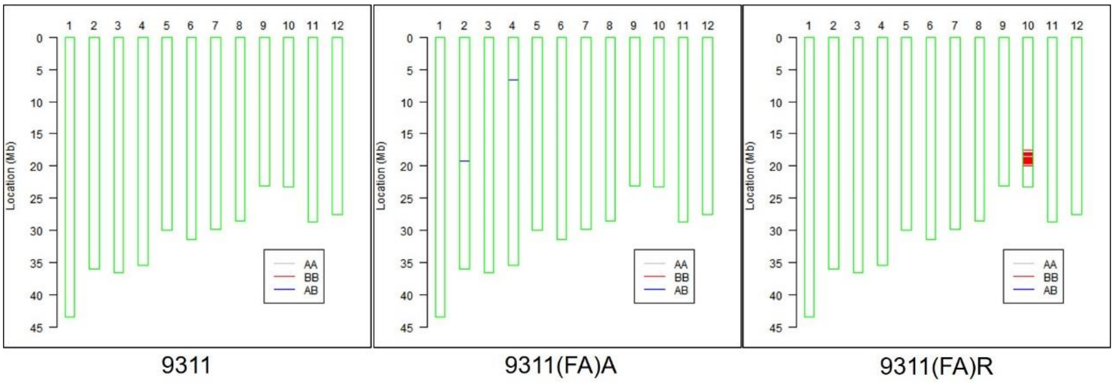

Fig. S6. Genotyping of 9311(FA)A and 9311(FA)R using the RICE6K microarray. 9311 is an elite cultivar widely grown in China. 9311(FA)A is a male sterile line with the 9311 nuclear genome and CMS-FA cytoplasm developed by successive backcrossing. 9311(FA)R is a restorer line having *OsRf19* in the 9311 genetic background.

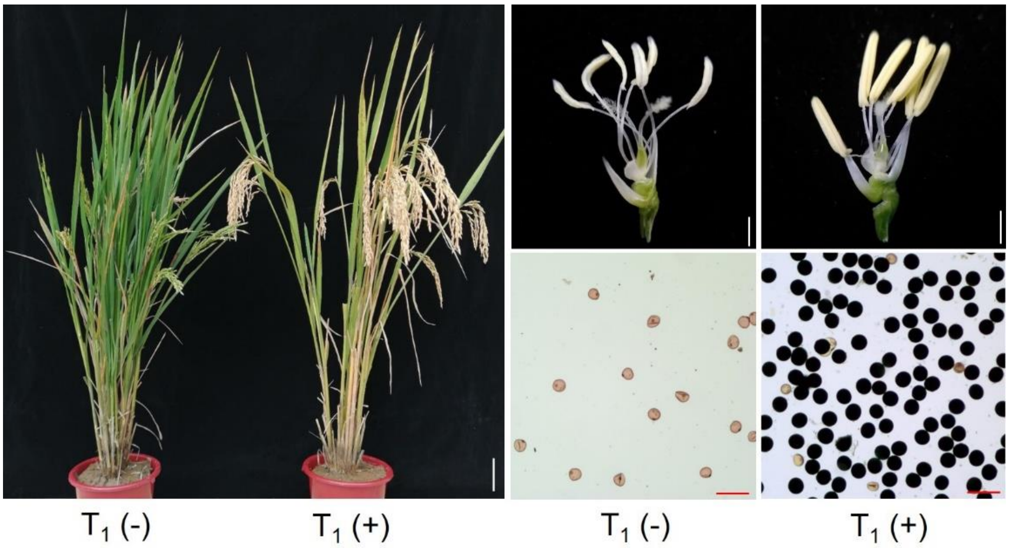

Fig. S7. Functional complementation of the construct containing *ORF1+ORF2*. Whole plant, anther and pollen grains of transgene-negative (-) and positive (+) T_1 plants at the maturation stage. Pollen grains are stained with 1% I_2-KI. Scale bars, 10 cm (plant); 1 mm (anthers); 50 µm (pollen).

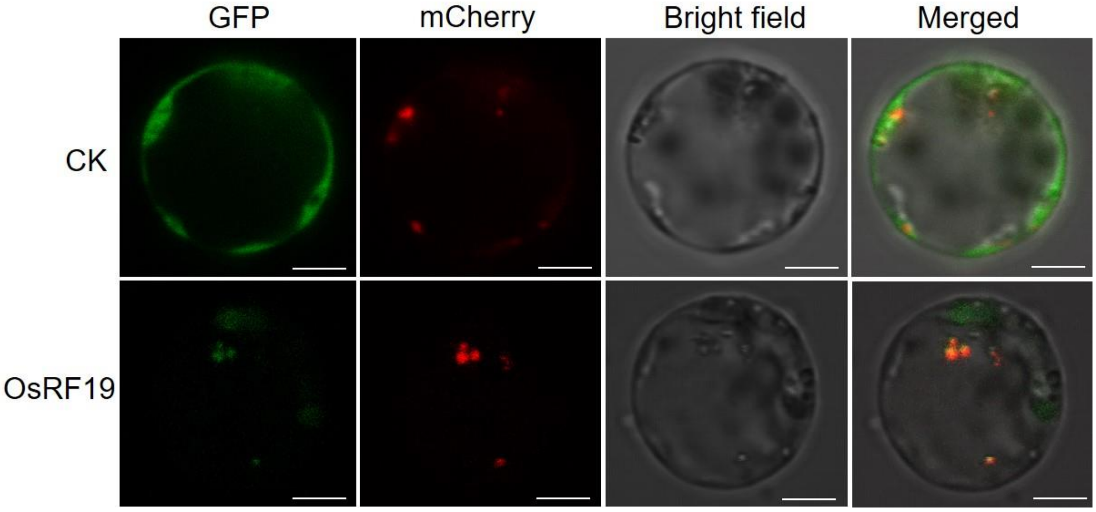

Fig. S8. Subcellular localization of OsRF19 in rice protoplasts. Co-localization of OsRF19-GFP and CD3-mCherry in mitochondria (bottom). Empty vectors pBI221-GFP and CD3-mCherry are co-transformed into rice protoplasts as a control (top). GFP, green fluorescent protein; mCherry, mCherry fluorescence protein (red). Scale bars, 5 μm.

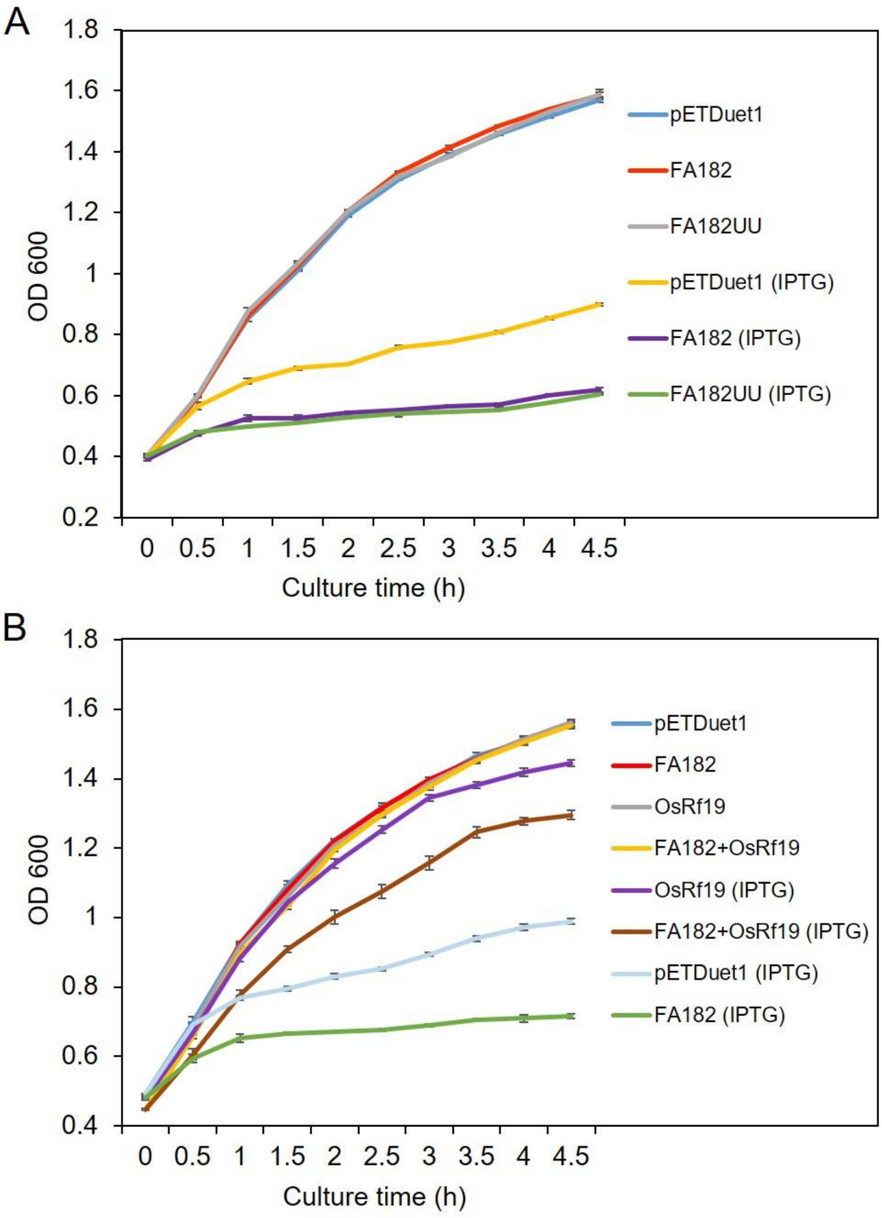

Fig. S9. Influence of *FA182* and *OsRf19* expression and co-expression on *E. coli* growth in cultures with or without IPTG. (*A*) Induced expression of *FA182* and *FA182UU* by IPTG in *E. coli* shows that FA182 and FA182UU are cytotoxic to bacteria, which hinders *E. coli* growth. (*B*) Co-expression of *FA182* and *OsRf19* in *E. coli* induced by IPTG shows that OsRF19 removes the cytotoxicity to bacteria caused by FA182 and that the bacteria continue to grow. The empty vector pETDuet1 was used as a control.

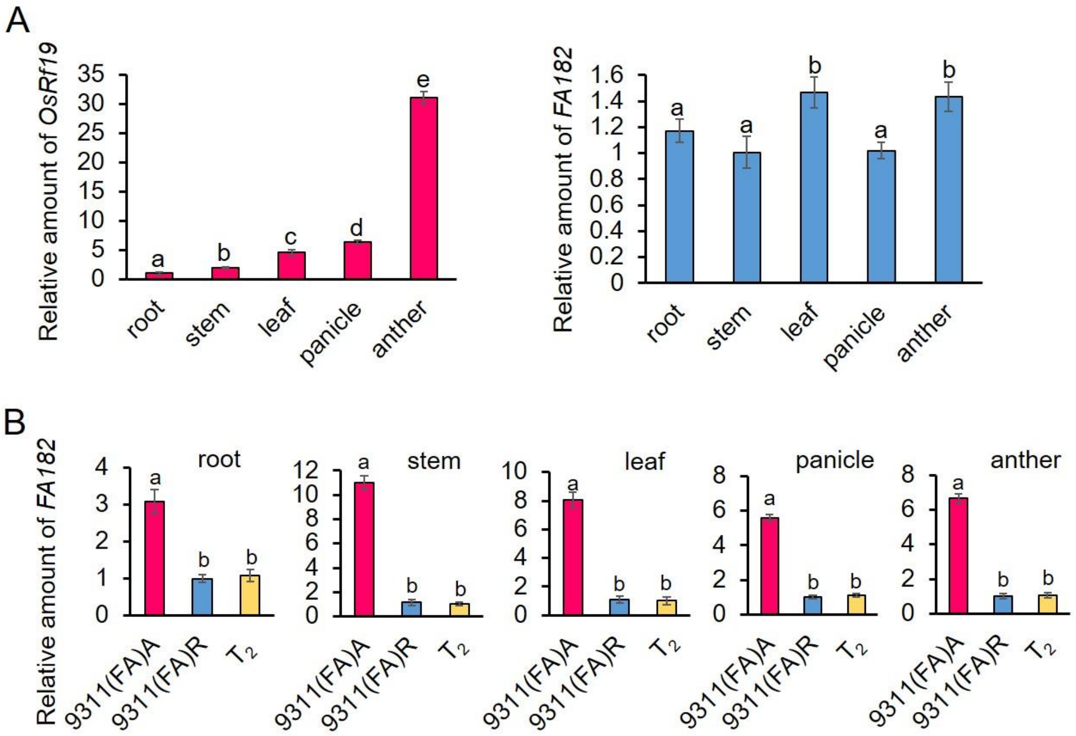

Fig. S10. Relative expression analysis of *FA182* in 9311(FA)A, 9311(FA)R and transgenic T_2 plants. (*A*) Relative expression analysis of *OsRf19* and *FA182* in roots, stems, leaves, panicles and anthers of 9311(FA)R and 9311(FA)A. Values are presented as the means ± SD (n = 3). Different letters denote significant differences determined by Tukey's tests, $P < 0.01$. (*B*) qRT-PCR analysis of the expression of *FA182* in root, stem, leaf, panicle and anther of 9311(FA)A, 9311(FA)R, and the transgenic T_2 plants using primer FA182-2F/2R. Values are presented as the means ± SD (n = 3). Different letters denote significant differences determined by Tukey's tests, $P < 0.01$.

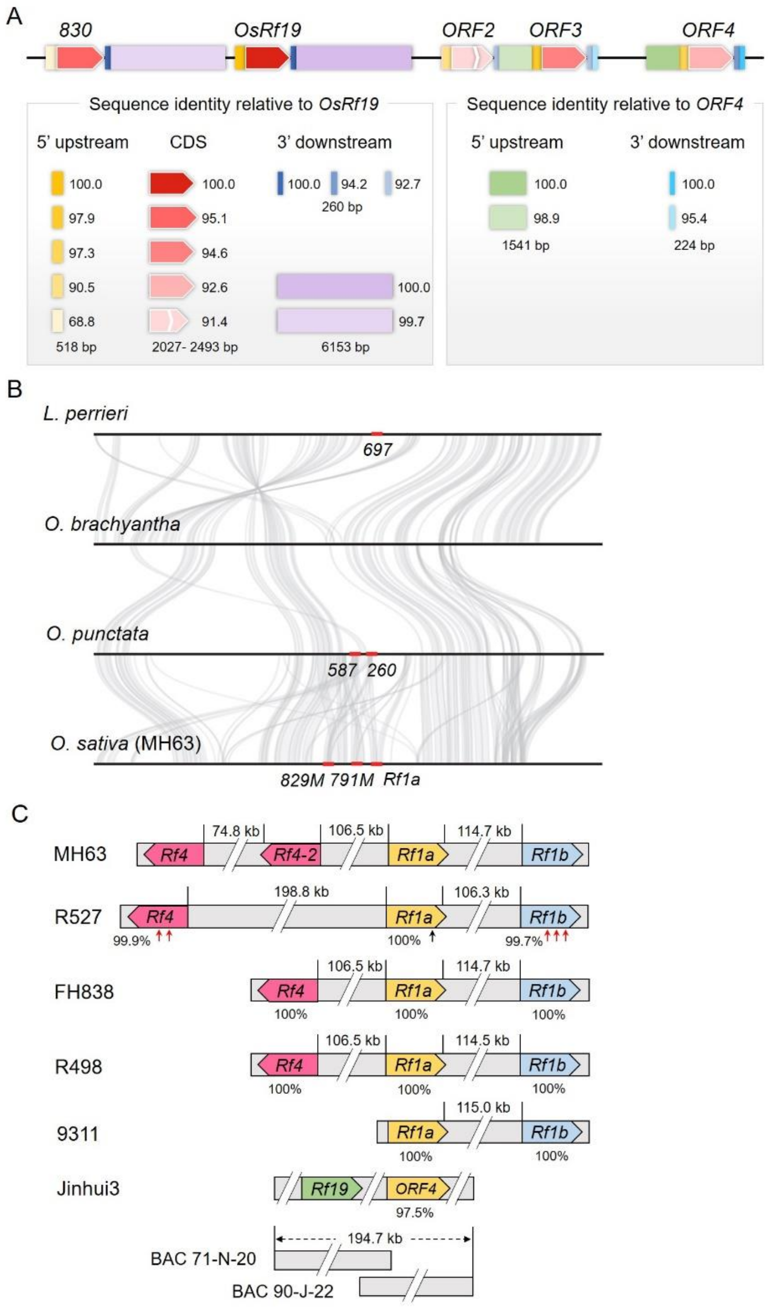

Fig. S11. Sequence analysis of the *OsRf19* region among different species and rice varieties. (*A*) Sequence analysis of the *OsRf19* region with five PPR genes in Jinhui3. (*B*) Synteny of the 200 kb *OsRf19* orthologous regions in *L. perrieri*, *O. brachyantha*, *O. punctata* and *O. sativa* (MH63). The orthologous sequences with identity higher than 80% are connected using gray lines. The *PPR* genes are indicated in red. (*C*) Cloned fertility restorer genes on chromosome 10 of rice restorer lines. Red arrows represent changes in amino acids, and black arrow indicates an insertion of T base. Sequence identity percentages are from the comparison of homologous genes with those in MH63.

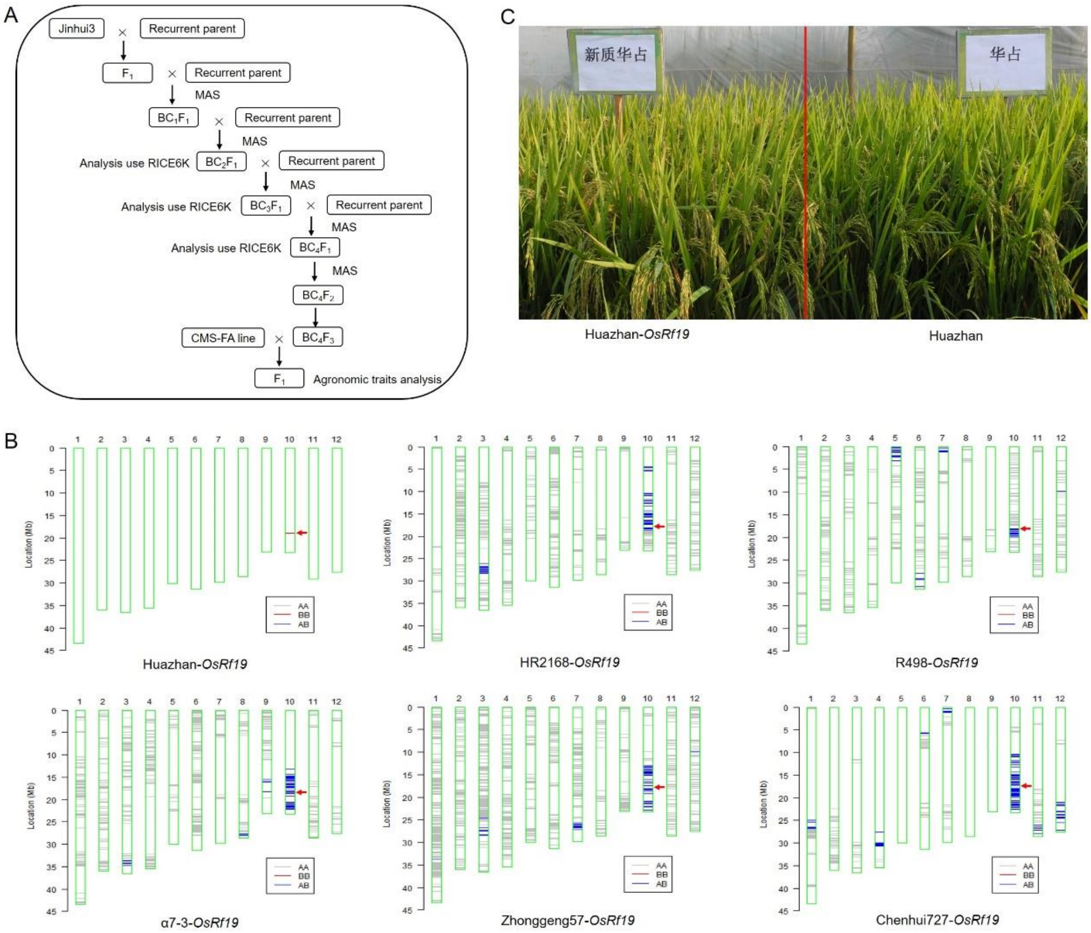

Fig. S12. Breeding application of the CMS-FA/*OsRf19* system. (*A*) Development of CMS-FA hybrids used in the breeding program. The breeding process involved four backcrosses and two generations of selfing. MAS, marker assisted selection. (*B*) Genetic background analysis of six derived restorer lines with *OsRf19* using the RICE6K microarray. The black, blue and red short lines on the chromosomes represent the single nucleotide polymorphism (SNP) sites with different genotypes. The red arrow indicates the positions of *OsRf19*. (*C*) Field performance of the derived restorer line Huazhan-*OsRf19* and the original parent Huazhan.

Table S1. Number of T_0 and T_1 transgenic plants of *FA182* and *FA182UU*.

Vectors	T_0 generation		T_1 generation	
	Plants with no or unstainable pollen grains	Plants with stainable pollen grains	Male fertility	Male sterility
35S-Rf1bMTS-*FA182*	9	6	42	40
Pubi-Rf1bMTS-*FA182*	5	4	39	36
Pubi-ATPγMTS-*FA182*	21	0	48	53
Pubi-ATPγMTS-*FA182UU*	16	0	35	37

35S, the CaMV35S promoter; Rf1bMTS, the mitochondrial transit peptide sequence was derived from the *OsRf1b* gene; Pubi, the maize ubiquitin promoter; ATP*γ*MTS, the mitochondrial transit peptide sequence was derived from the ATP*γ* gene. T_1 plants were obtained by crossing three male-sterile (plants with unstainable pollen grains) T_0 plants with wild-type Zhonghua11 plants.

Table S2. Genome variation analysis of *Oryza* genus accessions and *L. perrieri* at the *OsRf19* locus.

Genus	Species	Subspecies	Variety	Length (bp)	No. of PPR gene
Leersia	*L. perrieri*			undefined	1
Oryza	*O. brachyantha* (FF)			undefined	0
	O. punctata (BB)			8709	2
	O. meridionalis (AA)			19447	3
	O. glaberrima (AA)			13350	2
	O. barthii (AA)			13326	2
	O. sativa (AA)	*japonica*	Nipponbare	19694	3
			KY131	19694	3
			NamRoo	19693	3
			Kosh	19694	3
			ZH11	19694	3
			LJ	19693	3
			02428	19694	3
		indica-type1	Minghui 63	23160	3
			9311	23159	3
			S548	23158	3
			G46	23159	3
			CN1	23160	3
			FH838	23160	3
			G8	23160	3
			II32	23160	3
			TM	23160	3
			YX1	23160	3

			ZS97	23160	3
			Y58S	23159	3
			D62	23159	3
			J4155	23159	3
			DG	23160	3
			FS32	23160	3
			G630	23160	3
			R498	23160	3
			Y3551	23160	3
			Lemont	23130	3
			N22	23137	3
		indica-type2	IR24	27390	4
			WSSM	27394	4
			R527	27394	4
			Tumba	27739	4
			DHX2	24654	4
			IR64[*]	15216	2
			Basmati1[*]	15090	2
	O. rufipogon (AA)		Jinhu3 (target fragment from *O. rufipogon*)	37185	5
	O. nivara (AA)			47208	6

[*] The rice accessions were not used in Fig. 3.

Table S3. Agronomic performance of the restorer lines.

Variety	No. of days to heading	Plant height (cm)	No. of tillers per plant	No. of grains per panicle	Spikelet fertility (%)	1000-grain weight (g)	Yield per plant (g)
HR2168	102.7±1.3	102.3±1.9	9.8±1.7	124.5±18.1	79.5±6.1	26.2±2.2	25.0±4.9
HR2168-*OsRf19*	103.3±1.1	102.8±4.9	9.6±1.6	118.3±17.3	81.4±4.9	25.9±1.8	23.6±3.5
R498	100.3±0.5	103.5±3.2	7.7±1.9	155.6±29.7	81.7±6.0	30.1±1.3	29.2±8.9
R498-*OsRf19*	100.1±0.33	101.3±3.3	7.9±1.4	144.6±25.5	83.0±7.2	29.2±1.1	27.9±8.6
α7-3	93.3±0.5	92.9±2.2	9.0±1.3	138.9±17.1	88.1±4.3	24.8±0.9	27.2±5.5
α7-3-*OsRf19*	97.7±0.7**	96.5±2.6**	9.3±2.2	136.7±27.1	83.2±7.5	25.2±1.7	26.5±7.7
Zhonggeng57	95.2±0.9	90.7±3.2	6.8±1.2	219.0±60.2	76.2±6.4	21.9±2.0	24.3±6.9
Zhonggeng57-*OsRf19*	95.9±0.9	88.5±3.0	6.5±1.2	206.4±32.7	80.5±7.2	23.9±1.1	25.4±4.7
Chenghui727	103.8±0.8	99.8±1.8	8.9±1.8	127.6±14.6	78.1±4.5	28.0±1.9	25.2±7.1
Chenghui727-*OsRf19*	105.1±0.9	97.8±4.4	9.6±2.0	138.5±31.6	76.7±6.4	28.2±2.4	28.8±9.9

Values are presented as the means±SD.

Significant difference from the performance of the new *OsRf19* restorer lines when compared with the original parental lines at ** $P<0.01$.

Table S4. Agronomic performance of test-cross F_1s against a commercial elite hybrid.

Female	Male	No. of days to heading	Plant height (cm)	No. of tillers per plant	No. of grains per panicle	Spikelet fertility (%)	1000-grain weight (g)	Yield per plant (g)
Fengliangyou4 (CK)		92.2±0.4	128.5±4.5	8.8±2.0	191.6±22.6	75.16±5.7	25.01±0.6	32.0±9.4
Jinnong3(FA)A	Huazhan-*OsRf19*	93.8±0.4	125.2±6.7	10.8±2.1	206.1±24.8	80.72±6.1	19.06±0.6	35.1±12.3
Jinnong3(FA)A	HR2168-*OsRf19*	91.7±0.5	129.6±6.0	8.9±2.8	177.7±34.6	86.23±6.5	22.62±0.8	32.7±16.8
Jinnong3(FA)A	R498-*OsRf19*	94.9±0.3	130.2±4.7	7.8±2.6	196.7±33.0	87.32±4.4	23.81±0.5	32.4±14.7
Jinnong3(FA)A	α7-3-*OsRf19*	92.7±0.5	133.3±5.3	9.6±2.6	198.1±28.1	83.89±5.3	21.21±0.7	34.2±11.5
Jinnong3(FA)A	Chenhui727-*OsRf19*	93.9±0.3	134.0±7.9	8.3±2.4	194.8±39.3	83.75±7.0	23.67±0.9	33.4±14.9
Jinnong3(FA)A	Zhonggeng57-*OsRf19*	86.7±0.5	126.3±2.8	7.9±1.9	258.8±41.9	78.85±5.9	21.19±1.0	34.4±11.3
Jinnong3(FA)A	9311-*OsRf19*	94.1±0.3	125.7±7.7	8.4±2.1	193.8±37.7	87.29±4.7	23.31±0.8	32.4±8.1
Jinnong3(FA)A	Yuehui94-*OsRf19*	94.6±0.5	133.2±5.1	9.4±2.0	207.9±28.5	81.46±4.9	23.86±0.8	38.6±11.9**
Jinnong3(FA)A	Jinhui3	95.1±0.3	131.8±5.9	9.3±2.1	195.1±28.5	79.6±5.1	24.92±0.8	36.2±11.7**

Values are presented as the means±SD.

Significant difference from the performance of the F_1s when compared with the checks Fengliangyou4 at ** P<0.01.

Table S5. Grain yield of the hybrids in the field plot trial.

Female	Male	Yield (ton/ha)
Fengliangyou4 (CK)		10.8 ±0.4
Shen95(FA)A	Huazhan-*OsRf19*	10.9 ±0.5
Jinnong3(FA)A	Yuehui94-*OsRf19*	11.8 ±0.5
Jinnong3(FA)A	Jinhui3	12.3 ±0.6**

Values are presented as the means ±SD.

Significant difference from the performance of the F_1s when compared with the checks Fengliangyou4 at ** P<0.01.

Table S6. Primers used in this study.

**Markers used in mapping *OsRf19* and screening BAC clones**	
RM6100-F	TTCCCTGCAAGATTCTAGCTACACC
RM6100-R	TGTTCGTCGACCAAGAACTCAGG
Rf1D6-F	CTCTTCAAAATCGTCCAGG
Rf1D6-R	GTTGAAGGACCAGTATCTGG
Rf1D7-F	CGTCACCTTTGTCTTTCAC
Rf1D7-R	CGTACTAGCACAATCTATCATG
Rf1S57-F	AATACATCGTCGCCTCACAC
Rf1S57-R	GCAAGAGCAACAAGCAAAAG
RM171-F	ACGAGATACGTACGCCTTTG
RM171-R	ACGAGATACGTACGCCTTTG
Rf1D3-F	GTCCTTTAAGTTGCCTCTG
Rf1D3-R	GCTAAAGCGAAATTATTCTGTGC
Rf1D5-F	TCGTCCGTAAATCCGATTG
Rf1D5-R	GTGCGATAGCAGCGACATC
TMRf1M02-F	CCCTGCACAAACAGTGAACTTC
TMRf1M02-R	GGATGCTTCAGGTTCAGGACTT
TMRf1M02-1	CGGAAACAGAgAGCAAT
TMRf1M02-2	CGGAAACAGAaAGCAAT
TMRf1M06-F	GCAGTAGTTTTGGCATTAGATCCA
TMRf1M06-R	AACACCTAAACAGTACACAAATGAAATTT
TMRf1M06-1	GTAGGATTCATTTGTaTGTATC
TMRf1M06-2	GTAGGATTCATTTGTgTGTATC
TMRf1M10-F	CGATCAGATAATATCCGATCCAATC
TMRf1M10-R	TCGGACAAAAATGTCGGATAATC
TMRf1M10-1	TTTTTAGAAATCTGGcGGATAT
TMRf1M10-2	GTTTTTAGAAATCTGGtGGATAT
**Primers for detect CRISPR/Cas9 induced mutant of *OsRf19* gene**	
OsRf19-mutant-F	CAACCGGATCATCATGTATTCAA
OsRf19-mutant-R	CCTTCTTTGCAAAGATTGCTA
**Primers for construct expression vector of *FA182* and *OsRf19* in *E. coli***	
FA182-pETDuet1-F	ATCATCACCACAGCCAGGATCCGATGATGAGATTT AGTTCAAC
FA182-pETDuet1-R	GCGGCCGCAAGCTTGTCGACTCATGGGAACCACT TGCTGAAT
OsRf19-pETDuet1-F	GATATACATATGGCAGATCTGATGGCGCGCCGCGC CGCTT
OsRf19-pETDuet1-R	TTACCAGACTCGAGGGTACCGCAGCTCAAAGATT CTAT
**Primers for RT-PCR and qRT-PCR**	

FA182-1F	ATGAAACACTCCTACGGCAG
FA182-1R	CAGAGCGATACCATCAAGAT
FA182-2F	GATGAGATTTAGTTCAACGG
FA182-2R	GTCGGATGGACCAGCGGCCA
OsRf19-qRT-F	GATGTACTTTGCAAGTCAGGC
OsRf19-qRT-R	CCTTCTTTGCAAAGATTGCT
Ubq-F	ACCCTGGCTGACTACAACATC
Ubq-R	AGTTGACAGCCCTAGGGTG
**Primers for RLM-RACE assay**	
RNA adapter	GCUGAUGGCGAUGAAUGAACACUGCGUUUGCUG GCUUUGAUGAAA
RACE outer primer	GCTGATGGCGATGAATGAACACTG
Gene-specific primer 1	TCATGGGAACCACTTGCTGAAT
RACE inner primer	CGCGGATCCGAACACTGCGTTTGCTGGCTTTGATG
Gene-specific primer 2	TTGGTCAATAATTTGAAGCC
**Primers for RNA editing analysis**	
FA182-3F	ATGATGAGATTTAGTTCAAC
FA182-3R	CTAGAATAGAACGGATGGTG

Dataset S1 (separate file). PPR genes in orthologous regions of the *OsRf19* locus.

References

1. J. M. Chen *et al.*, Substoichiometrically different mitotypes coexist in mitochondrial genomes of *Brassica napus* L. *PLoS One* **6,** e17662 (2011).
2. D. Antipov, A. Korobeynikov, J. S. Mclean, P. A. Pevzner, HYBRIDSPADES: an algorithm for hybrid assembly of short and long reads. *Bioinformatics* **32,** 1009–1015 (2016).
3. M. Goel, H. Sun, W. B. Jiao, K. Schneeberger, SyRI: finding genomic rearrangements and local sequence differences from whole-genome assemblies. *Genome Biol.* **20,** 277 (2019).
4. Y. M. Yu, Y. D. Ouyang, W. Yao, shinyCircos: an R/Shiny application for interactive creation of Circos plot. *Bioinformatics* **34,** 1229–1231 (2017).
5. Z. H. Wang *et al.*, Cytoplasmic male sterility of rice with Boro II cytoplasm is caused by a cytotoxic peptide and is restored by two related PPR motif genes via distinct modes of mRNA silencing. *Plant Cell* **18,** 676–687 (2006).
6. K. Wang *et al.*, ORFH79 impairs mitochondrial function via interaction with a subunit of electron transport chain complex III in Honglian cytoplasmic male sterile rice. *New Phytol.* **198,** 408–418 (2013).
7. N. Y. Wang *et al.*, Development of CMS-FA hybrid rice combination Jinnong 2 You 3. *J. Fujian Agric. Forestry Univ.* **43,** 345–349 (2014) (In Chinese with English abstract).
8. H. H. Yu *et al.*, A whole-genome SNP array (RICE6K) for genomic breeding in rice. *Plant Biotechnol. J.* **12,** 28–37 (2014).
9. X. Shi, H. Y. Zeng, Y. D. Xue, M. Z. Luo, A pair of new BAC and BIBAC vectors that facilitate BAC/BIBAC library construction and intact large genomic DNA insert exchange. *Plant Methods* **7,** 33 (2011).
10. X. L. Ma *et al.*, A robust CRISPR/Cas9 system for convenient, high-efficiency multiplex genome editing in monocot and dicot plants. *Mol. Plant* **8,** 1274–1284 (2015).
11. B. K. Nelson, X. Cai, A. Nebenfuhr, A multicolored set of in vivo organelle markers

for co-localization studies in Arabidopsis and other plants. *Plant J.* **51,** 1126–1136 (2007).

12. Y. Zhang *et al.*, A highly efficient rice green tissue protoplast system for transient gene expression and studying light/chloroplast-related processes. *Plant Methods* **7,** 30 (2011).
13. K. Maruyama, S. Sugano, Oligo-capping: a simple method to replace the cap structure of eukaryotic mRNAs with oligoribonucleotides. *Gene* **138,** 171–174 (1994).
14. B. C. Shaefer, Revolution in rapid amplification of cDNA ends: new strategies for polymerase chain reaction cloning of full-length cDNA ends. *Analytical Biochem.* **227,** 255–273 (1995).
15. K. J. Livak, T. D. Schmittgen, Analysis of relative gene expression data using real-time quantitative PCR and the $2^{-\Delta\Delta Ct}$ method. *Methods* **25,** 402–408 (2001).
16. T. Komori *et al.*, Map-based cloning of a fertility restorer gene, *Rf-1*, in rice (*Oryza sativa* L.). *Plant J.* **37,** 315–25 (2004).
17. P. Qin *et al.*, Pan-genome analysis of 33 genetically diverse rice accessions reveals hidden genomic variations. *Cell* **184,** 3542–3558 (2021).
18. J. C. Stein *et al.*, Genomes of 13 domesticated and wild rice relatives highlight genetic conservation, turnover and innovation across the genus *Oryza*. *Nat. Genet.* **50,** 285–296 (2018).
19. J. M. Song *et al.*, Two gap-free reference genomes and a global view of the centromere architecture in rice. *Mol. Plant* **14,** 1757–1767 (2021).
